# Empagliflozin lowers serum uric acid in chronic kidney disease: exploratory analyses from the EMPA-KIDNEY trial

**DOI:** 10.1093/ndt/gfae203

**Published:** 2024-09-14

**Authors:** Kaitlin J Mayne, Rebecca J Sardell, Natalie Staplin, Parminder K Judge, Doreen Zhu, Emily Sammons, David Z I Cherney, Jennifer B Green, Adeera Levin, Roberto Pontremoli, Sibylle J Hauske, Jonathan Emberson, David Preiss, Martin J Landray, Colin Baigent, Christoph Wanner, Richard Haynes, William G Herrington, the EMPA-KIDNEY Collaborative Group, Colin Baigent, Martin J Landray, Christoph Wanner, William G Herrington, Richard Haynes, Jennifer B Green, Sibylle J Hauske, Martina Brueckmann, Mark Hopley, Maximillian von-Eynatten, Jyothis George, Alfred K Cheung, Zhi-Hong Liu, Jing Li, Laiseong Hooi, Wen Liu, Takashi Kadowaki, Masaomi Nangaku, Adeera Levin, David Cherney, Roberto Pontremoli, Aldo P Maggioni, Natalie Staplin, Stefan Hantel, Shinya Goto, Rajat Deo, Katherine R Tuttle, Parminder Judge, Kaitlin J Mayne, Sarah Y A Ng, Xavier Rossello, Emily Sammons, Doreen Zhu, Peter Sandercock, Rudolf Bilous, Charles Herzog, Paul Whelton, Janet Wittes, Derrick Bennett

**Affiliations:** Renal Studies Group, Clinical Trial Service Unit and Epidemiological Studies Unit, Nuffield Department of Population Health, University of Oxford, Oxford, UK; School of Cardiovascular and Metabolic Health, University of Glasgow, Glasgow, UK; Renal Studies Group, Clinical Trial Service Unit and Epidemiological Studies Unit, Nuffield Department of Population Health, University of Oxford, Oxford, UK; Renal Studies Group, Clinical Trial Service Unit and Epidemiological Studies Unit, Nuffield Department of Population Health, University of Oxford, Oxford, UK; Renal Studies Group, Clinical Trial Service Unit and Epidemiological Studies Unit, Nuffield Department of Population Health, University of Oxford, Oxford, UK; Oxford Kidney Unit, Oxford University Hospitals NHS Foundation Trust, Oxford, UK; Renal Studies Group, Clinical Trial Service Unit and Epidemiological Studies Unit, Nuffield Department of Population Health, University of Oxford, Oxford, UK; Oxford Kidney Unit, Oxford University Hospitals NHS Foundation Trust, Oxford, UK; Renal Studies Group, Clinical Trial Service Unit and Epidemiological Studies Unit, Nuffield Department of Population Health, University of Oxford, Oxford, UK; University of Toronto, Toronto, ON, Canada; Duke Clinical Research Institute, Durham, NC, USA; University of British Columbia, Vancouver, BC, Canada; Università degli Studi and IRCCS Ospedale Policlinico San Martino di Genova, Genova, Italy; Boehringer Ingelheim International GmbH, Ingelheim am Rhein, Germany & Vth Department of Medicine, University Medical Center Mannheim, University of Heidelberg, Mannheim, Germany; Renal Studies Group, Clinical Trial Service Unit and Epidemiological Studies Unit, Nuffield Department of Population Health, University of Oxford, Oxford, UK; Renal Studies Group, Clinical Trial Service Unit and Epidemiological Studies Unit, Nuffield Department of Population Health, University of Oxford, Oxford, UK; Renal Studies Group, Clinical Trial Service Unit and Epidemiological Studies Unit, Nuffield Department of Population Health, University of Oxford, Oxford, UK; Renal Studies Group, Clinical Trial Service Unit and Epidemiological Studies Unit, Nuffield Department of Population Health, University of Oxford, Oxford, UK; Renal Studies Group, Clinical Trial Service Unit and Epidemiological Studies Unit, Nuffield Department of Population Health, University of Oxford, Oxford, UK; Renal Studies Group, Clinical Trial Service Unit and Epidemiological Studies Unit, Nuffield Department of Population Health, University of Oxford, Oxford, UK; Oxford Kidney Unit, Oxford University Hospitals NHS Foundation Trust, Oxford, UK; Renal Studies Group, Clinical Trial Service Unit and Epidemiological Studies Unit, Nuffield Department of Population Health, University of Oxford, Oxford, UK; Oxford Kidney Unit, Oxford University Hospitals NHS Foundation Trust, Oxford, UK

**Keywords:** CKD, empagliflozin, gout, SGLT2 inhibitor, uric acid

## Abstract

**Background:**

Hyperuricaemia and gout are common in chronic kidney disease (CKD). We aimed to assess the effects of sodium–glucose co-transporter-2 (SGLT2) inhibition on uric acid (urate) and gout in patients with CKD.

**Methods:**

The EMPA-KIDNEY trial randomised 6609 patients with CKD to receive either empagliflozin 10 mg daily or matching placebo over a median of 2 years of follow-up. Serum uric acid was measured at randomisation then at 2 and 18 months of follow-up and the effects of empagliflozin were analysed using a pre-specified mixed model repeated measures approach. Participant-reported gout events were analysed in Cox regression models (first events) with the Andersen–Gill extension (total events). A post hoc composite outcome included new initiation of uric acid–lowering therapy or colchicine. EMPA-KIDNEY primary and kidney disease progression outcomes were also assessed in subgroups of baseline serum uric acid.

**Results:**

Baseline mean ± standard deviation serum uric acid concentration was 431 ± 114 µmol/l. Allocation to empagliflozin resulted in a study-average between-group difference in serum uric acid of −25.6 µmol/l [95% confidence interval (CI) −30.3 to −21.0], with larger effects in those with higher eGFR (trend *P* < .001) and without diabetes (heterogeneity *P* < .001). Compared with placebo, empagliflozin did not significantly reduce first or total gout events [hazard ratio 0.87 (95% CI 0.74–1.02) for the 595 first events and 0.86 (0.72–1.03) for the 869 total events] with similar hazard ratios for the post hoc composite and across subgroups, including by diabetes and eGFR. The effect of empagliflozin on the primary outcome and kidney disease progression outcomes were similar irrespective of the baseline level of uric acid.

**Conclusions:**

SGLT2 inhibition reduces serum uric acid in patients with CKD, with larger effects at higher eGFR and in the absence of diabetes. However, the effect on uric acid is modest and did not translate into reduced risk of gout in EMPA-KIDNEY.

## Graphical Abstract

**Graphical Abstract:**
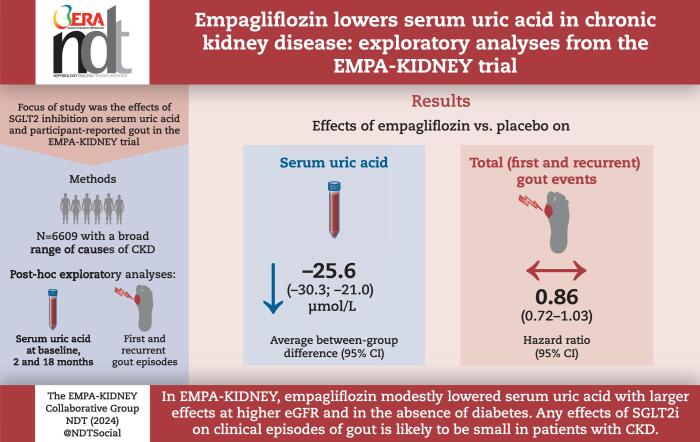

KEY LEARNING POINTS **What was known:**

- Hyperuricaemia and gout are common in chronic kidney disease (CKD).
- Sodium–glucose co-transporter-2 (SGLT2) inhibitors reduce uric acid and may reduce the risk of gout in people with diabetes and/or heart failure.
- Uric acid–lowering effects at lower levels of kidney function were uncertain.

**This study adds:**

- SGLT2 inhibition modestly lowers uric acid in CKD, with greater effects at higher levels of kidney function and in the absence of diabetes (with particular attenuation when glycaemic control is poor).
- This uric acid–lowering effect does not appear to explain the protective effects of SGLT2 inhibition on the kidney.

**Potential impact:**

- Uric acid lowering may be an additional advantage of SGLT2 inhibitor treatment in patients with CKD. The magnitude of this effect is small compared with uricosuric agents, but SGLT2 inhibitors further lower uric acid in addition to such therapies in patients with CKD.

## INTRODUCTION

Uric acid is derived from metabolism of dietary purines (such as meat, fish, seafood and alcohol) as well as endogenous production via the pentose phosphate pathway, which supports the synthesis of fatty acids, nucleotides and aromatic amino acids [1]. Excretion of uric acid is largely via the kidneys [1]. Uric acid is freely filtered by the glomerulus then reabsorbed in the proximal tubules by the urate transporter URAT1 and the facilitative hexose/urate transporter GLUT9b [1]. Decreased glomerular filtration leads to increased serum uric acid concentration [2] and an increased prevalence of gout in chronic kidney disease (CKD) [2]. Hyperuricaemia is also associated with the risk of further CKD progression [1, 2], but the association has not been proven to be causal in Mendelian randomisation analyses [3–5] or in randomised trials of interventions that lower uric acid [6–8].

Sodium–glucose co-transporter-2 (SGLT2) inhibitors have been demonstrated in large randomised controlled trials to slow the progression of kidney disease in adults with CKD by >30% and to reduce acute kidney injury by ≈25% [9]. Additionally, SGLT2 inhibitors are known to reduce uric acid levels in patients with diabetes and/or heart failure [10–14], yet randomised clinical trial evidence in patients with CKD is lacking. This is important due to the high prevalence of hyperuricaemia and because low estimated glomerular filtration rate (eGFR) may modify the effects of uric acid lowering with SGLT2 inhibitors. We aimed to quantify the effect of empagliflozin on serum uric acid levels in the EMPA-KIDNEY trial (NCT03594110) population and relate any uric acid lowering to any effects on the risk of episodes of gout and CKD progression.

## MATERIALS AND METHODS

The full methods of the EMPA-KIDNEY trial and the main results have been reported elsewhere (ClinicalTrials.gov) [15]. Briefly, patients with CKD at risk of progression were identified based on historical and screening local laboratory measurements of an eGFR ≥20 but <45 ml/min/1.73 m^2^ or an eGFR ≥45 but <90 ml/min/1.73 m^2^ with a urinary albumin:creatinine ratio (uACR) ≥200 mg/g. All participants provided written informed consent. Regulatory authorities and ethics committees in eight participating countries approved the trial.

### Outcomes

Serum uric acid was measured at randomisation and at 2 and 18 months of follow-up in all participants who gave consent for long-term sample storage for biochemical analysis. Participants were asked about their history of gout at baseline and any occurrences of gout (serious and non-serious) at every follow-up visit.

A post hoc composite outcome combining first occurrences of gout with new initiation of uric acid–lowering therapy or colchicine was constructed for participants not already taking such therapy at randomisation. Uric acid–lowering therapy was ascertained from concomitant medication reports on routine visit forms at randomisation and at each follow-up visit. Uric acid–lowering therapy included purine (allopurinol) and non-purine (febuxostat and topiroxostat) xanthine oxidase inhibitors and the primary uricosuric agents benzbromarone and probenecid. There were no recorded uses of oxypurinol, tisopurine, phytic acid, myo-inositol or sulfinpyrazone in EMPA-KIDNEY. Analyses did not include the use of non-steroidal anti-inflammatory drugs, steroids or interleukin inhibitors, as these are not specific to the treatment of gout.

The primary composite outcome in EMPA-KIDNEY was time to first occurrence of progression of kidney disease [defined as end-stage kidney disease (ESKD), the initiation of maintenance dialysis or receipt of a kidney transplant, a sustained decrease in eGFR to <10 ml/min/1.73 m^2^, a sustained decrease from baseline in eGFR of ≥40% or death from kidney failure] or death from cardiovascular causes [15]. Kidney disease progression was a secondary outcome and both outcomes were assessed in post hoc exploratory subgroup analyses according to baseline serum uric acid levels.

### Statistical analysis

The effects of empagliflozin on serum uric acid were analysed using a pre-specified mixed model repeated measures (MMRM) approach adjusted for age, sex, region, eGFR, uACR and diabetes status in the categories pre-specified for the minimised randomisation algorithm. The MMRM also included fixed categorical effects of time (to avoid assuming a linear association between treatment allocation and uric acid over time), treatment allocation, treatment × time interaction and continuous effects of baseline (randomisation) uric acid, and baseline × time interaction. The within-person error correlations were assumed to be unstructured. Effects at each follow-up time point were estimated and used to derive study-average effects (with weights proportional to the amount of time between visits). All between-group differences are reported as empagliflozin minus placebo.

For time-to-first-event outcomes, the effects of allocation to empagliflozin versus placebo were assessed using pre-specified Cox regression models adjusted for age, sex, region, eGFR, uACR and diabetes status. Total (first and recurrent) gout events were analysed using the Andersen–Gill extension of Cox regression. For subgroup analyses by baseline serum uric acid level, participants were categorised according to approximate thirds of the variable's distribution, with a missing category analysed separately but included in analyses of the full trial population.

To assess effect modification, subgroup-specific treatment effects were estimated by fitting interaction terms in both the MMRM and Cox regression model and calculating a heterogeneity or trend statistic from subgroup-specific estimates and standard errors, without formal correction for multiplicity of testing. The null hypothesis was that the treatment effect is the same across all subgroups. Important subgroups pre-selected for these analyses were sex, diabetes, baseline eGFR, baseline serum uric acid, prior history of gout, diuretic therapy and uric acid–lowering therapy or colchicine reported at baseline. Additional subgroups were developed to explore effects according to insulin use and by glycated haemoglobin at baseline after identifying evidence of heterogeneity by diabetes status.

In mediation analyses, the proportion of treatment effect on chronic eGFR slope (from 2 months to the end of follow-up; calculated for each individual participant using linear regression) explained by on-study serum uric acid levels was estimated using the landmark method, adjusting the linear regression model for serum uric acid measured at the 2-month (post-randomisation) study visit. The proportion of treatment effect explained by biomarkers relates to the percentage reduction in the Wald χ^2^ statistic when the biomarker variable or combination of biomarker variables is added to the model. This reflects the reduction in the strength of the association between treatment allocation and chronic eGFR slope after adjustment for the biomarker(s). This analysis was restricted to participants with non-missing uric acid (and other biomarkers) at 2 months and a minimum of two eGFR measurements between 2 months and the final follow-up visit. Bias-corrected and accelerated bootstrap intervals with 10 000 replications were used to construct the 95% confidence intervals (CIs). Analyses were performed using R Studio version 4.2.2 (Posit PBC, Boston, MA) and SAS version 9.4 (SAS Institute, Cary, NC, USA).

## RESULTS

### Baseline characteristics

Baseline serum uric acid was measured at randomisation in 5168 of 6609 randomised participants (78%). The mean ± standard deviation (SD) serum uric acid concentration was 431 ± 114 µmol/l (Table 1; Supplementary Tables 1 and 2) and similar in males and females (433 ± 114 versus 428 ± 112 µmol/l). Higher body mass index, a history of heart failure and use of diuretic therapy were more prevalent among those with hyperuricaemia (Table 1). Higher baseline serum uric acid was also associated with a greater 5-year risk of kidney failure (using the Kidney Failure Risk Equation [16]) (Table 1), however, the correlation between serum uric acid and eGFR was weak (Supplementary Fig. 1). Serum uric acid at randomisation in those allocated to placebo was strongly positively associated with the future risk of an episode of gout (Supplementary Fig. 2).

**Table 1:** Characteristics of participants at baseline by serum uric acid concentration at randomisation.

	Serum uric acid at randomisation, µmol/l^a^			
Characteristics	<380 (*n* = 1753)	≥380–<470 (*n*= 1691)	≥470 (*n* = 1724)	Missing^b^ (*n* = 1441)
Demographics				
Age at randomisation (years), mean (SD)	67.6 (12.0)	66.1 (12.9)	63.8 (14.2)	56.7 (14.0)
Sex, *n* (%)				
Male	1160 (66.2)	1124 (66.5)	1175 (68.2)	958 (66.5)
Female	593 (33.8)	567 (33.5)	549 (31.8)	483 (33.5)
Race (all regions), *n* (%)				
White	1225 (69.9)	1154 (68.2)	1187 (68.9)	293 (20.3)
Black	66 (3.8)	85 (5.0)	88 (5.1)	23 (1.6)
Asian	437 (24.9)	423 (25.0)	421 (24.4)	1112 (77.2)
Mixed	4 (0.2)	7 (0.4)	6 (0.3)	4 (0.3)
Other	21 (1.2)	22 (1.3)	22 (1.3)	9 (0.6)
Prior disease, *n* (%)				
Diabetes^c^	867 (49.5)	836 (49.4)	850 (49.3)	487 (33.8)
Cardiovascular disease^d^	519 (29.6)	455 (26.9)	547 (31.7)	244 (16.9)
Heart failure	181 (10.3)	173 (10.2)	241 (14.0)	63 (4.4)
Gout	568 (32.4)	416 (24.6)	456 (26.5)	267 (18.5)
Cause of kidney disease, *n* (%)				
Diabetic kidney disease	568 (32.4)	556 (32.9)	588 (34.1)	345 (23.9)
Hypertension/renovascular	393 (22.4)	383 (22.6)	430 (24.9)	239 (16.6)
Glomerular	343 (19.6)	345 (20.4)	351 (20.4)	630 (43.7)
Other/unknown	449 (25.6)	407 (24.1)	355 (20.6)	227 (15.8)
Clinical measurements				
Blood pressure (mmHg)				
Systolic, mean (SD)	135.4 (18.2)	136.6 (18.0)	136.6 (18.2)	137.7 (18.6)
Diastolic, mean (SD)	76.6 (11.3)	77.6 (11.4)	76.8 (12.2)	81.9 (11.6)
Body mass index (kg/m^2^), mean (SD)	29.7 (6.5)	30.2 (6.8)	31.9 (7.1)	26.7 (5.4)
Laboratory measurements				
eGFR (ml/min/1.73 m^2^)				
Mean (SD)	38.8 (14.7)	36.4 (12.9)	33.4 (11.6)	41.3 (17.3)
<30	507 (28.9)	599 (35.4)	774 (44.9)	402 (27.9)
≥30–<45	812 (46.3)	778 (46.0)	741 (43.0)	597 (41.4)
≥45	434 (24.8)	314 (18.6)	209 (12.1)	442 (30.7)
uACR (mg/g)				
Geometric mean (95% CI)	186 (169–204)	189 (171–208)	179 (163–197)	434 (398–474)
Median (IQR)	261 (35–987)	273 (35–992)	257 (34–944)	572 (201–1383)
<30	407 (23.2)	386 (22.8)	401 (23.3)	134 (9.3)
≥30–≤300	526 (30.0)	482 (28.5)	511 (29.6)	345 (23.9)
>300	820 (46.8)	823 (48.7)	812 (47.1)	962 (66.8)
NT-proBNP (ng/l)				
Geometric mean (95% CI)	184 (173–196)	186 (174–198)	210 (196–225)	105 (98–113)
Median (IQR)	170 (75–443)	175 (76–427)	195 (80–533)	107 (49–244)
Concomitant medication use, *n* (%)				
RAS inhibitor	1441 (82.2)	1445 (85.5)	1504 (87.2)	1238 (85.9)
Any diuretic therapy	711 (40.6)	741 (43.8)	1057 (61.3)	306 (21.2)
Loop diuretic	454 (25.9)	457 (27.0)	684 (39.7)	152 (10.5)
Thiazide diuretic	275 (15.7)	296 (17.5)	410 (23.8)	141 (9.8)
Lipid-lowering therapy	1260 (71.9)	1224 (72.4)	1273 (73.8)	621 (43.1)
Any uric acid lowering/gout therapy	974 (55.6)	552 (32.6)	279 (16.2)	545 (37.8)
Xanthine oxidase inhibitor^e^	968 (55.2)	543 (32.1)	257 (14.9)	447 (31.0)
Primary uricosuric agent^f^	10 (0.6)	5 (0.3)	5 (0.3)	98 (6.8)
Colchicine	39 (2.2)	30 (1.8)	37 (2.1)	5 (0.3)
Smoking and alcohol				
Ever smoked tobacco regularly^g^	859 (49.0)	805 (47.6)	821 (47.6)	464 (32.2)
Ever drank alcohol regularly^h^	834 (47.6)	795 (47.0)	729 (42.3)	316 (21.9)
5-year risk of kidney failure (%), median (IQR)	6.6 (2.3–21.0)	8.4 (2.8–27.4)	12.8 (4.8–37.3)	10.8 (2.6–31.6)

a To convert uric acid to mg/dl, divide by 59.48 (380 µmol/l ≈ 6.4 mg/dl; 470 µmol/l ≈ 7.9 mg/dl).

b Reasons for missing uric acid: analyses were not conducted in participants from China (*n* = 986) and long-term sample storage for biochemical analysis required additional optional consent.

c Defined as participant-reported history of diabetes of any type, use of glucose-lowering medication or baseline HbA1c ≥48 mmol/mol at randomisation visit.

d Defined as participant-reported history of myocardial infarction, heart failure, stroke, transient ischaemic attack or peripheral arterial disease.

e Allopurinol, febuxostat or topiroxostat.

f Benzbromarone or probenecid.

g Defined as most days for at least 1 year.

h Defined as at least 1 day a week for at least 1 year.

NT-proBNP: N-terminal pro-B-type natriuretic peptide; RAS: renin–angiotensin system.

Participants reporting previous gout had lower mean ± SD serum uric acid (422 ± 121 versus 435 ± 110 µmol/l) and were more likely to be taking uric acid–lowering therapy or colchicine at baseline (Supplementary Table 2). There was considerable overlap between participant groups with a self-reported history of gout, those on uric acid–lowering/colchicine treatment and with evidence of hyperuricaemia (defined as uric acid ≥470 µmol/l; Supplementary Fig. 3). Nevertheless, the use of uric acid–lowering medication or colchicine was common in the absence of a history of self-reported gout; of 2350 participants prescribed such medications at baseline, 1179 (50%) did not report prior gout (Supplementary Table 1; indications for their use among those without gout are unknown).

### Effects of empagliflozin on serum uric acid

**Figure 1:**
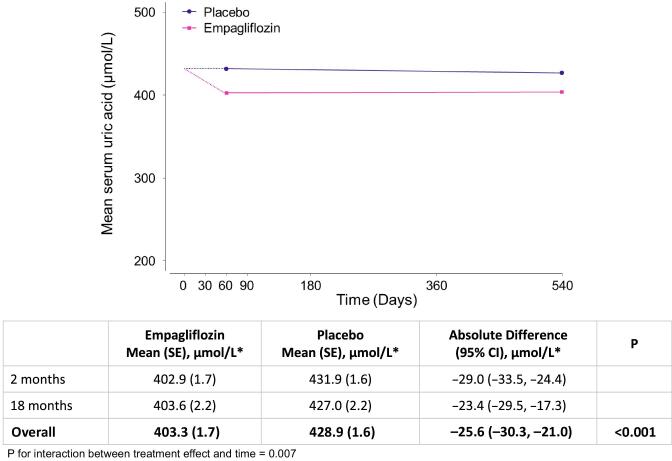
Effect of empagliflozin versus placebo on serum uric acid overall. *To convert uric acid to mg/dl, divide by 59.48 (25.6 µmol/l ≈ 0.4 mg/dl). Analysis required participants to have at least one measurement of uric acid during follow-up at 2 and/or 18 months (*n* = 2691 participants). The value at time 0 is the overall mean of baseline values in all analysed participants in the empagliflozin and placebo arms combined. Study-average differences between treatment groups (empagliflozin minus placebo) are derived from an MMRM adjusted for baseline serum uric acid (in continuous form), baseline × time interaction, the covariates used in the minimisation algorithm (categories of age, sex, diabetes, eGFR, uACR and region), fixed categorical effects of time, treatment allocation and treatment × time interaction and weighted in proportion to the amount of time between follow-up visits. Missing baseline uric acid (4/2691 participants) was imputed with the baseline mean.

**Figure 2:**
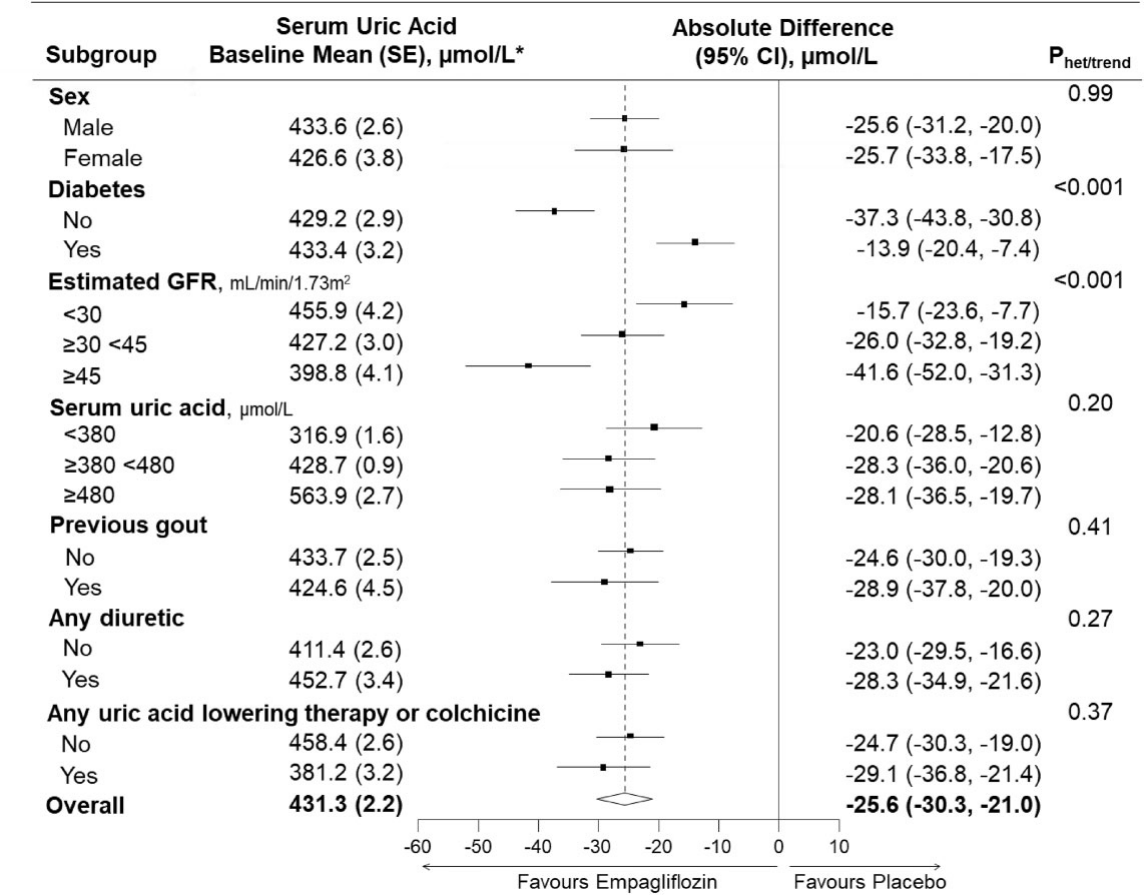
Effects of empagliflozin versus placebo on serum uric acid in subgroups defined by baseline characteristics. *To convert uric acid to mg/dl, divide by 59.48 (380 µmol/l ≈ 6.4 mg/dl; 480 µmol/l ≈ 8.1 mg/dl). Analysis required participants to have at least one measurement of uric acid during follow-up at 2 and/or 18 months (*n* = 2691 participants). Absolute differences in study-average uric acid between treatment groups (empagliflozin minus placebo) are derived from an MMRM adjusted for baseline serum uric acid (in continuous form), baseline × time interaction, the covariates used in the minimisation algorithm (categories of age, sex, diabetes, eGFR, uACR and region), fixed categorical effects of time, treatment allocation and treatment × time interaction and weighted in proportion to the amount of time between follow-up visits. Interaction terms are included in the MMRM to assess for heterogeneity (sex, diabetes, previous gout, any diuretic therapy) between or trend (eGFR, serum uric acid) across subgroup-specific means and standard errors. Relative differences are presented in Supplementary Fig. 4. Uric acid–lowering therapy includes xanthine oxidase inhibitors and primary uricosuric drugs (see Methods). Missing baseline uric acid (4/2691 participants) was imputed with the baseline mean and thus participants are included in the middle subgroup category (≥380–<480 µmol/l).

In 2691 participants who had at least one uric acid measurement during follow-up, empagliflozin resulted in a significant reduction in serum uric acid levels that occurred by 2 months and were sustained until at least 18 months (Fig. 1). The between-group difference (empagliflozin minus placebo) averaged over 18 months was −25.6 µmol/l (95% CI −30.3 to −21.0). The observed uric acid–lowering effect was similar in both males and females and was unmodified by baseline uric acid levels, prior history of gout and diuretic therapy use or uric acid–lowering/colchicine therapy use at baseline (all heterogeneity/trend *P*-values >.10) (Fig. 2 and Supplementary Fig. 4). Uric acid–lowering effects were larger among those with a higher level of eGFR at baseline (trend *P* < .001; Fig. 2 and Supplementary Figs. 4–6). Furthermore, the effect of empagliflozin on uric acid was larger in those without diabetes than those with diabetes (heterogeneity *P* < .001; Fig. 2 and Supplementary Figs. 4–6) and the magnitude of difference between those with versus without diabetes was itself greater at higher versus lower levels of eGFR.

### Effects of empagliflozin on gout and clinically important hyperuricaemia

**Figure 3:**
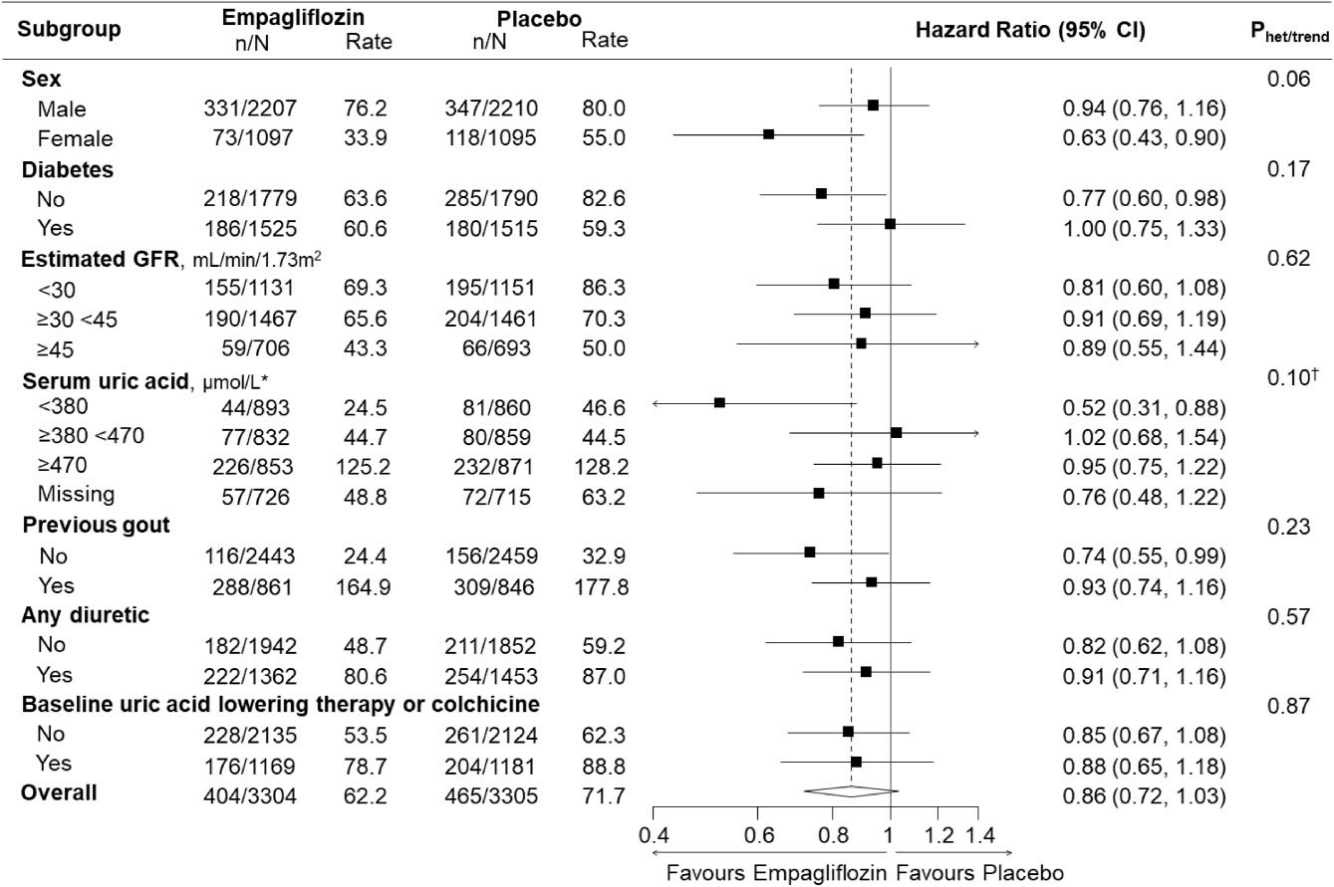
Effects of empagliflozin versus placebo on first and recurrent gout events in subgroups. Total (first and recurrent) self-reported episodes of gout recorded as adverse events (serious or non-serious) analysed using the Andersen–Gill extension of Cox regression with adjustment for the covariates used in the minimisation algorithm (categories of age, sex, diabetes, eGFR, uACR and region). *n*/*N* represents the number of events/the number of participants and rate is expressed as the total events per 1000 patient-years. Reasons for missing uric acid: analyses were not conducted in participants from China (*n* = 986) or participants who did not provide additional optional consent for long-term sample storage for biochemical analysis. *To convert uric acid to mg/dl, divide by 59.48 (380 µmol/l ≈ 6.4 mg/dl; 470 µmol/l ≈ 7.9 mg/dl). ^†^Trend test excludes missing category. Uric acid–lowering therapy includes xanthine oxidase inhibitors and primary uricosuric drugs (see Methods).

There were 869 adverse event reports of gout throughout the median 2.0-year (IQR 1.5–2.4) follow-up in 595 participants (404 events in 278 participants in the empagliflozin group and 465 events in 317 participants in the placebo group). Overall, compared with placebo, empagliflozin did not significantly reduce the occurrence of first or total (first and recurrent) gout events [hazard ratio (HR) 0.87 (95% CI 0.74–1.02) and 0.86 (0.72–1.03), respectively; Table 2]. HRs were broadly similar in subgroup analyses of time to any gout event (first and recurrent), including analyses by baseline eGFR (trend *P* = .62) and diabetes status (heterogeneity *P* = .17; Fig. 3). The HR was also similar in a post hoc analysis excluding 2350 participants already taking uric acid–lowering therapy or colchicine at randomisation [first gout event or initiation of uric acid–lowering or colchicine therapy: 261 events in 2135 participants allocated to empagliflozin (12.2%) versus 314/2124 (14.8%) in the placebo group; HR 0.81 (95% CI 0.69–0.96)].

**Table 2:** Effects of empagliflozin versus placebo on gout.

	Empagliflozin		Placebo		
	*n*/*N*	Rate per 1000 patient-years	*n*/*N*	Rate per 1000 patient-years	HR (95% CI)
First occurrence of gout^a^	278/3304	45.7	317/3305	52.3	0.87 (0.74–1.02)
All occurrences of gout^b^	404/3304	62.2	465/3305	71.7	0.86 (0.72–1.03)

All analyses use a time-to-first-event approach. Cox proportional hazards models include adjustment for the covariates used in the minimisation algorithm (categories of age, sex, diabetes, eGFR, uACR and region).

In a post hoc analysis, excluding participants not already taking uric acid–lowering therapy or colchicine at randomisation, empagliflozin resulted in a 19% reduction in the hazards of the composite of a first gout event or initiation of uric acid–lowering or colchicine therapy [HR 0.81 (95% CI 0.69–0.96); 261 events in the empagliflozin group versus 314 in the placebo group).

a Self-reported episode of gout recorded as an adverse event (serious or non-serious); previously reported (EMPA-KIDNEY Collaborative Group, 2023).

b First and recurrent events analysed using the Andersen–Gill extension of Cox regression.

### Effects of empagliflozin on the primary outcome and kidney disease progression according to baseline serum uric acid

**Figure 4:**
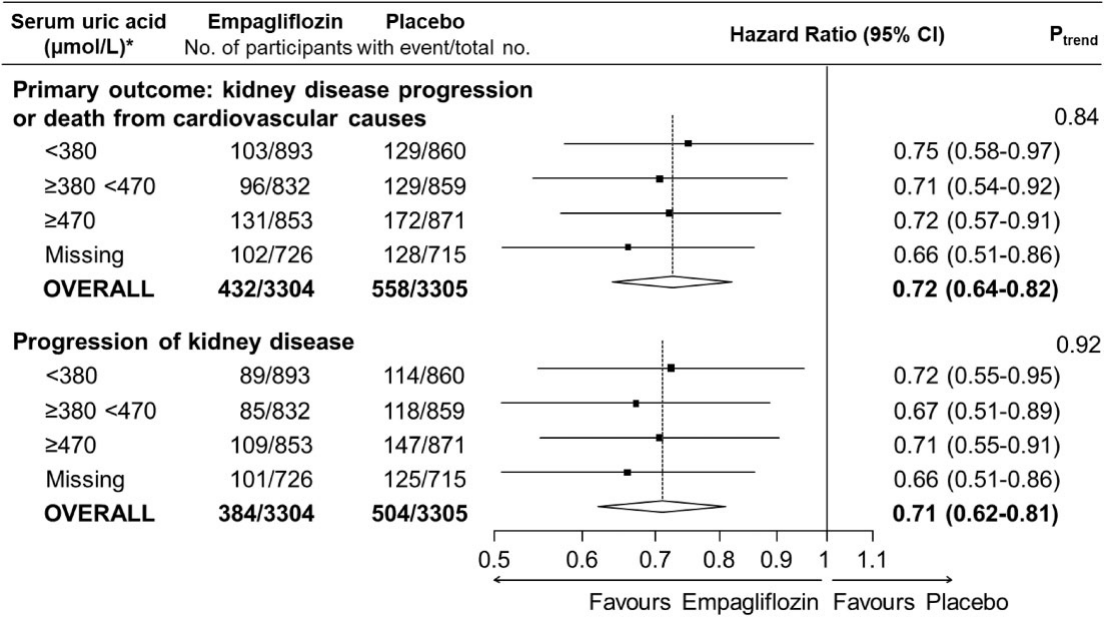
Effects of empagliflozin versus placebo on the primary outcome and kidney disease progression by serum uric acid concentration at randomisation. *To convert uric acid to mg/dl, divide by 59.48 (380 µmol/l ≈ 6.4 mg/dl; 470 µmol/l ≈ 7.9 mg/dl). Progression of kidney disease is defined as ESKD (initiation of maintenance dialysis or kidney transplant), a sustained decrease in eGFR to <10 ml/min/1.73 m^2^, a sustained decrease in eGFR of at least 40% from baseline or death from renal causes. Reasons for missing uric acid: analyses were not conducted in participants from China (*n* = 986) or participants who did not provide additional optional consent for long-term sample storage for biochemical analysis. *P*_trend_ tests for trend across the three non-missing categories.

As previously reported, in the full EMPA-KIDNEY population and compared with placebo, empagliflozin reduced the risk of the primary composite outcome of kidney disease progression or cardiovascular death by 28% [HR 0.72 (95% CI 0.64–0.82)]. There was no significant difference in this relative effect by baseline serum uric acid level (trend *P* = .84; Fig. 4). The majority of the 990 primary outcome events were due to kidney disease progression (888 events) and, overall, empagliflozin reduced the risk of this secondary outcome by 29% [HR 0.71 (95% CI 0.62–0.81)], again with no significant heterogeneity according to serum uric acid concentration at randomisation (trend *P* = .92; Fig. 4).

### Mediation of the effects of empagliflozin on chronic eGFR slope by serum uric acid

In exploratory analyses, there was no evidence that the effect of empagliflozin versus placebo on chronic eGFR slope (nor the primary outcome or key secondary outcome of kidney disease progression) was explained by on-study (i.e. measurement from the 2-month visit) serum uric acid. On-study uACR and systolic and diastolic blood pressure explained 15% (95% CI 10–22%), 6% (3–11%) and 1% (0–4%) of the treatment effect on chronic eGFR slope, respectively. When combined with haemoglobin A1c (HbA1c) and uric acid, these variables together still only explained 15% (95% CI 7–23%) of the effect of empagliflozin on chronic eGFR slope (Supplementary Table 3).

## DISCUSSION

These exploratory analyses sought to quantify the effect of SGLT2 inhibition on uric acid and gout in a broad population of patients with CKD at risk of progression. In EMPA-KIDNEY, empagliflozin resulted in an early and sustained reduction in serum uric acid, with larger effects at higher eGFR and in those without diabetes. In exploratory analyses, the beneficial effects of empagliflozin on kidney disease progression were not dependent upon baseline uric acid level nor were they explained by on-study uric acid levels. Overall, the average effect was relatively modest (a 6% relative reduction in uric acid levels) and did not translate into a measurable effect on clinical risk of gout events in this trial population. This effect of SGLT2 inhibitors on uric acid is much smaller than that achieved by xanthine oxidase inhibitors (≈30–50%) [17] or the newer URAT1 inhibitor verinurad (≈40–60%) [8, 18]. Nevertheless, empagliflozin lowered serum uric acid in participants reporting such uric acid–lowering therapy use at baseline (Fig. 2), suggesting a mechanism of action not saturated by existing therapies and likely complementary effects.

The uric acid–lowering effect observed in EMPA-KIDNEY is within the range of that reported from other large SGLT2 inhibitor trials. These effects vary widely with a study-average between-group difference ranging from 13 µmol/l in CREDENCE to ≈50–70 µmol/l in the DAPA-HF, EMPEROR-Reduced and EMPEROR-Preserved trials [10, 12, 19, 20]. Our analyses raise a hypothesis that apparent differences in uric acid–lowering effects between the large SGLT2 inhibitor trials may reflect differences in trial population characteristics, specifically relating to diabetes and the level of kidney function. For example, uric acid–lowering effects are greatest in the heart failure trials composed of ≈50% of participants with and without diabetes and relatively preserved kidney function (Supplementary Table 4) and smallest in a CKD trial that exclusively recruited participants with type 2 diabetes where mean glycated haemoglobin was 8.3% and two-thirds were on insulin at baseline [19, 21]. Post hoc analyses of the EMPEROR-Reduced trial also suggest effect modification by diabetes status: the adjusted mean difference in serum uric acid with empagliflozin versus placebo was −1.25 mg/dl (95% CI −1.36 to −1.14) compared with −0.99 mg/dl (95% CI −1.09 to −0.88) in those without and with diabetes, respectively (≈74 and 59 µmol/l; heterogeneity *P* < .001) [12]. The differential uric acid–lowering effects we observed between those with and without diabetes in EMPA-KIDNEY are not entirely explained by differences in baseline eGFR (or vice versa; Supplementary Fig. 5).

The mechanisms by which empagliflozin lowers serum uric acid could encompass both enhanced renal excretion and reduced synthesis of uric acid [23]. SGLT2 inhibition increases tubular glucose, which competitively antagonises urate reabsorption via the GLUT9b transporter in the proximal tubule [1]. However, SGLT2 inhibitor-driven uricosuria cannot solely be explained by glycosuria, as we observed larger effects in participants without diabetes where glycosuria is attenuated [24]. Animal studies suggest uricosuria may additionally be enhanced via direct effects of SGLT2 inhibitors on urate transporters in the kidney (GLUT9 and ABCG2) [25, 26]. The phenomenon of attenuated uric acid lowering in patients with diabetes might be explained by antagonist effects of insulin in modulating urate transporters in the kidney, which has been demonstrated in animal studies [27]. Outside of the kidney, SGLT2 inhibitors may also reduce uric acid synthesis via downregulation of the pentose phosphate pathway (i.e. the source of purines that are metabolised to uric acid) [23]. An additional consideration is that SGLT2 inhibitors can reduce the need for initiation of additional diuretic therapy [28]—as was also observed in EMPA-KIDNEY (Supplementary Fig. 4 footnote)—likely due to reductions in total body water [29]. This is relevant since loop and thiazide diuretics reduce uric acid excretion, promote hyperuricaemia and are associated with an increased risk of gout [30, 31]. Our exploratory observations should encourage further mechanistic research into the interrelationship between glycaemic control, insulin, kidney function and the uric acid–lowering effects of SGLT2 inhibitors.

We found no evidence that the effect of empagliflozin on chronic eGFR slope (nor the composite outcome of kidney disease progression) was explained by on-study uric acid levels. This is consistent with Mendelian randomisation experiments in which genetic scores for uric acid levels do not predict progression of CKD [3–5]. Our findings are also consistent with mediation analyses from the CREDENCE trial, which was also conducted in a population with CKD, but in contrast to reports from trials among populations with relatively preserved kidney function (Supplementary Table 4) [34–36]. It is possible that changes in uric acid reflect changes in kidney function more closely in such populations (where creatinine-based formulae perform poorly), so the apparent mediation was confounded by kidney function. An interesting observation concerning the relationship between eGFR and the uric acid–lowering effect of empagliflozin is that the reduction in uric acid occurred early (Fig. 1) despite the expected acute dip in eGFR [15, 22]. Changes in uric acid can also reflect weight loss [37–39], but since the ≈1-kg effective weight loss in empagliflozin-treated patients in EMPA-KIDNEY is entirely explained by fluid loss (and not fat) [29], weight loss would not be expected to explain the uric acid–lowering observed in EMPA-KIDNEY.

A strength of these analyses (despite only using three measurement time points) was the large number of participants with uric acid measurements. This provides precise estimates of effects in subgroups even though the magnitude of uric acid lowering by SGLT2 inhibitors was only ≈25 µmol/l in EMPA-KIDNEY (or ≈6% in relative terms). Such a small effect on uric acid may be too modest to detect any significant effect of SGLT2 inhibition on gout events even in large trials (Table 2). There is some evidence from early trials that gout events are reduced when available data are combined [32] and in analyses exploring post hoc outcomes based on initiation of uric acid–lowering therapy [11–13, 33]. In EMPA-KIDNEY, serum uric acid concentration was strongly positively associated with adverse event reports of gout (Supplementary Fig. 2). If this association is causal, the overall uric acid–lowering effect (≈25 µmol/l) of empagliflozin could be expected to reduce the risk of a first gout event by ≈7% (for a patient with a mean baseline uric acid value of ≈430 µmol/l as observed in EMPA-KIDNEY; Supplementary Fig. 2). Although the reported HRs for the clinical outcomes are consistent with a 7% reduction in relative risk, we estimate a few thousand gout episodes would be required for sufficient power to detect such an effect. Therefore, the main limitation of the present analyses—other than their post hoc exploratory nature—is that they were underpowered to assess whether the effect of empagliflozin on uric acid translates into effects on clinical episodes of gout. Reliance upon participant reports to ascertain gout events may be considered a limitation in EMPA-KIDNEY. However, direct follow-up by systematic interview instead of medical note review is our preferred method of follow-up, particularly for a symptomatic disease. This is because medical records used in routine clinical care are not recorded in a systematic manner and introduce error-prone transcription steps. Provided a trial is large, double-blind and placebo-controlled with systematic follow-up (i.e. it controls systematic bias), then small or even moderate amounts of missing data or data errors do not materially affect reported relative risks and only modestly affect power [40].

In conclusion, in EMPA-KIDNEY, empagliflozin modestly lowered serum uric acid in a broad range of patients with CKD, with larger effects at higher eGFR and in those without diabetes. There was minimal uric acid lowering in those with poor glycaemic control. Serum uric acid did not explain the effect of empagliflozin in slowing kidney disease progression. Since the effect of SGLT2 inhibition on uric acid is modest, any direct effect on clinical episodes of gout is likely to be small in patients with CKD.

## Supplementary Material

gfae203_Supplemental_File

## Data Availability

The complete de-identified patient dataset used for presented analyses will be available in due course. Departmental policy details can be found at https://www.ndph.ox.ac.uk/data-access. In adherence with the Boehringer Ingelheim Policy on Transparency and Publication of Clinical Study Data, scientific and medical researchers can request access to clinical study data, typically, 1 year after approval has been granted by major regulatory authorities or after termination of the development program. Researchers should use the https://vivli.org/ link to request access to study data and visit https://www.mystudywindow.com/msw/datasharing for further information.

## Notes

*See Supplementary Methods for full membership of the EMPA-KIDNEY Collaborative Group.
