# Supplementary material for: Empagliflozin lowers serum uric acid in chronic kidney disease: exploratory analyses from the EMPA-KIDNEY trial
Source: Nephrol Dial Transplant. 2024 Sep 14;40(4):720–30. doi: 10.1093/ndt/gfae203 (PMC7616479; doi:10.1093/ndt/gfae203)
Supplement: gfae203_Supplemental_File [file gfae203_Supplemental_File.pdf]

Empagliflozin lowers serum uric acid in chronic kidney disease: exploratory analyses from the EMPA-KIDNEY trial

Supplementary Material

Contents

Members of the EMPA-KIDNEY Collaborative Group 2
Membership of the Executive Committee, Steering Committee and Independent Data Monitoring Committee 2
Central and Regional Coordinating Centres 2
Central Coordinating Office 2
Regional Coordinating Centres 3
List of Collaborators, by Site 3
Supplementary Tables & Figures 11
Supplementary Table 1: Characteristics of participants at baseline in those taking uric acid lowering therapy or colchicine at randomisation 11
Supplementary Table 2: Characteristics of participants at baseline in those reporting a history of gout at randomisation 13
Supplementary Table 3: Proportion of the effect of empagliflozin on chronic eGFR slope explained by serum uric acid and other biomarkers at 2 months 15
Supplementary Table 4: Summary of uric acid lowering effects in other large SGLT2 inhibitor trials 16
Supplementary Figure 1: Correlation between serum uric acid & estimated glomerular filtration rate at randomisation by sex 17
Supplementary Figure 2: Relationship between baseline serum uric acid and first gout events in those allocated to placebo 18
Supplementary Figure 3: Relationship between history of previous gout; uric acid lowering therapy or colchicine; and hyperuricaemia at randomisation 19
Supplementary Figure 4: Absolute and relative effects of empagliflozin versus placebo on serum uric acid in subgroups defined by baseline characteristics 20
Supplementary Figure 5: Effects of empagliflozin versus placebo on serum uric acid by diabetes status and eGFR 21
Supplementary Figure 6: Further exploration of differential effects of empagliflozin on serum uric acid in participants with and without diabetes 22

Members of the EMPA-KIDNEY Collaborative Group

Membership of the Executive Committee, Steering Committee and Independent Data Monitoring Committee

Executive Committee
Colin Baigent (co-Chair), Martin J. Landray (co-Chair), Christoph Wanner (Deputy Chair), William G. Herrington (Chief Investigator), Richard Haynes (co-Principal Investigator), Jennifer B. Green, Sibylle J. Hauske*, Martina Brueckmann*, Mark Hopley*
(Previous members: Maximillian von-Eynatten* & Jyothis George*)

Steering Committee
Executive Committee members plus National Representatives: Susanne Brenner (Germany); Alfred K. Cheung (United States); David Preiss (United Kingdom); Zhi-Hong Liu, Jing Li (China); Laiseong Hooi, Wen Liu (Malaysia); Takashi Kadowaki, Masaomi Nangaku (Japan); Adeera Levin, David Cherney (Canada); Roberto Pontremoli, Aldo P. Maggioni (Italy); plus statistician members: Natalie Staplin, Jonathan Emberson, Stefan Hantel*; plus other expert members: Shinya Goto, Rajat Deo, Katherine R. Tuttle. Non-voting members: Michael Hill, Parminder Judge, Kaitlin J. Mayne, Sarah Y.A. Ng, Xavier Rossello, Emily Sammons, Doreen Zhu
* denotes a Boehringer Ingelheim employee

Independent Data Monitoring Committee
Peter Sandercock (Chair), Rudolf Bilous, Charles Herzog, Paul Whelton, Janet Wittes, Derrick Bennett (non-voting statistician)

Central and Regional Coordinating Centres

Central Coordinating Office

Administration: Patricia Achiri, Chrissie Ambrose, Cristina Badin, Jill Barton, Richard Brown, Andy Burke, Sebastian Butler, Rejive Dayanandan, Pia Donaldson, Robert Dykas, Lucy Fletcher, Kate Frederick, Hannah Kingston, Mo Gray, Emily Harding, Akiko Hashimoto, Lyn Howie, Susan Hurley, Ryonfa Lee, Nik Luker, Kevin Murphy, Mariko Nakahara, John Nolan, Michelle Nunn, Sorcha Mulligan, Akiko Omata, Sandra Pickworth, YanRu Qiao, Shraddha Shah, Karen Taylor, Alison Timadjer, Monique Willett, Liz Wincott, Qin Yan, Hui Yu; *Clinical*: Louise Bowman, Fang Chen, Robert Clarke, Michelle Goonasekera, Richard Haynes, William G. Herrington, Parminder Judge, Waseem Karsan, Marion Mafham, Kaitlin J. Mayne, Sarah Y. A. Ng, David Preiss, Christina Reith, Emily Sammons, Mohammed Zayed, Doreen Zhu; *Data Analysis*: Ritva Ellison, Rowan Moys, Will Stevens, Kevin Verdel, Karl Wallendszus; *Finance*: Chris Bowler, Anna Brewer, Andy Measor; *IT Validation*: Guanguo Cui, Charles Daniels, Angela Field, Bob Goodenough, Ashley Lawson, Youcef Mostefai, Dheeptha Radhakrishnan, Samee Syed, Shuang Xia; *Laboratory*: Ruth Adewuyi-Dalton, Thomas Arnold, Anne-Marie Beneat, Anoushka Bhatt, Chloe Bird, Andrew Breach, Laura Brown, Mark Caple, Tatyana Chavagnon, Karen Chung, Sarah Clark, Luminita Condurache, Katarzyna Eichstadt, Marta Espino Obrero, Scarlett Forest, Helen French, Nick Goodwin, Andrew Gordon, Joanne Gordon, Cat Guest, Tina Harding, Michael Hill, Michal Hozak, Matthew Lacey, David MacLean, Louise Messinger, Stewart Moffat, Martin Radley, Claire Shenton, Sarah Tipper, Jon Tyler, Lesley Weaving, James Wheeler, Elissa Williams, Tim Williams, Hamish Woodhouse; *Monitoring*: Angela Chamberlain, Jo Chambers, Joanne Davies, Denise Donaldson, Pati Faria-Shayler, Denise Fleming-Brown, Jennifer Ingell, Carol Knott, Anna Liew, Helen Lochhead, Juliette Meek, Isabel Rodriguez-Bachiller, Andrea Wilson, Patrick Zettergren; *Programming*: Rach AitSadi, Ian Barton, Alex Baxter, Yonghong Bu, Lukasz Danel, Sonja Grotjahn, Rijo Kurien, Michael Lay, Archie Maskill, Aleksandra

Murawska, Rachel Raff, Allen Young; *Principal Investigators*: Colin Baigent, Richard Haynes, William G. Herrington, Martin J. Landray, David Preiss; *Statistics*: Jonathan Emberson, Rebecca Sardell, Natalie Staplin

Regional Coordinating Centres

Germany (Universitätsklinikum Würzburg): Christoph Wanner, Susanne Brenner, Vladimir Cejka, Marcela Fajardo-Moser, Christian Hartner, Doris Poehler, Janina Renner, Franziska Scheidemantel

United States (Duke Clinical Research Institute [DCRI]): Jennifer B. Green, Miya Bryant, Anita Hepditch, Cassandra Johnson, Erin Latore, Yolanda Miller, Lauren Price, Merilee Whalen, Ashleigh Wheeler

UK (Clinical Trial Service Unit and Epidemiological Studies Unit [CTSU], University of Oxford): Richard Haynes, David Preiss, Cristina Badin, Jo Chambers, Joanne Davies, Denise Donaldson, Mo Gray, Emily Harding, Jenny Ingell, Yanru Qiao, Shraddha Shah, Andrea Wilson, Patrick Zettergren

China (National Center for Cardiovascular Disease, Fuwai Hospital & National Clinical Research Center of Kidney Diseases, Jinling Hospital, Nanjing University School of Medicine): Zhi-Hong Liu, Jing Li, Yu An, Yinghua Chen, Peiling Chen, Hao Dai, Hong Du, Fang Feng, Qing Guo, Libo Hou, Wuhanbilige Hundei, Binbin Jin, Yan Li, Jiamin Liu, Xia Song, Yanping Wang, Yanwu Yu, Ning Zhang, Lingshan Zhao, Hui Zhong

Malaysia (Klinsel SDN BHD): Cheng Beng Goh, Ye Mun Low, Soon Yi Sor, Farah Hanis Zulkipli, Sarojini Sivanandam

Japan (Parexel): Natsuki Arai, Ai Fukasawa, Mizue Furukawa, Keisuke Habuki, Shoko Hayashi, Wakako Isari, Saki Kanegae, Maria Kawai, Reiki Kobayashi, Takako Kuramae, Chika Kuribayashi, Sawako Maeno, Satoshi Masumoto, Tomoko Morisaki, Minoru Oda, Kazue Sawada, Kenta Sugamori, Ayana Tatsuzawa, Aiko Tomita, Kazuyuki Yuasa, Hiroko Inazawa

Canada (Providence Health Care, Vancouver): Adeera Levin, Amanda Axler, Kerri Gallo

Italy (ANMCO Research Center): Aldo P. Maggioni, Ester Baldini, Barbara Bartolomei Mecatti, Francesca Bianchini, Martina Ceseri, Laura Cipressa, Gianna Fabbri, Andrea Lorimer, Donata Lucci

List of Collaborators, by Site

Germany: *Universitatsklinikum Wurzburg*: Christoph Wanner, Susanne Brenner, Vladimir Cejka, Sharang Ghavampour, Anja Knoppe; *Zentrum fuer Nieren-, Hochdruck- und Stoffwechselerkrankungen Hannover*: Hans Schmidt-Gurtler, Hubert Dumann, Sybille Merscher, Margret Patecki, Georg Rainer Schlieper, Anke Torp, Bianca Weber, Maja Zietz; *Nephrologisches Zentrum Villingen-Schwenningen*: Bernd Hohenstein, Urs Benck, Diliana Draganova, Thomas Weinreich, Lothar Wolf, Jasmine Gaidu, Hanna Reiner, Mandy Visnjic; *Nierenzentrum Freiburg*: Daniel Steffl, Marie Breitenfeldt, Annette Kraemer-Guth, Christine Braun, Simone Hagge*; Dialysezentrum Heilbronn*: Michael Schomig, Stephan Matthias, Dominik Stoffler, Beate Schumacher; *Klinikum der Universitat Munchen*: Thomas Sitter, Louise Fuessl, Julia Krappe, Jerome Loutan, Volker Vielhauer, Luciano Andriaccio, Magdalena Maurer; *ClinPhenomics GmbH Co. KG*: Bernhard Winkelmann, Martin Dursch,

Linda Seifert, Linda Tenbusch; *Universitatsmedizin Mainz*: Julia Weinmann-Menke, Simone Boedecker, Wiebke Kaluza-Schilling, Daniel Kraus, Carina Krieger, Margit Schmude, Anne Schreiber, Ewelina Eckrich; *Herz- und Diabeteszentrum Nordrhein-Westfalen*: Diethelm Tschope, Abdulwahab Arbi, Young Lee-Barkey, Bernd Stratmann, Natalie Prib, Sina Rolfsmeier, Irina Schneider; *Universitatsklinikum Dusseldorf*: Lars Rump, Johannes Stegbauer, Christine Pötz, Mara Schemmelmann, Claudia Schmidt; *Nephrocare Mettmann - Standort Velbert*: Michael Koch, Sendogan Aker, Annika Küpper, Manuela Martin; *Diaverum MVZ Potsdam*: Thiemo Pfab, Christian Albert, Michael Haase, Barbara Zander, Claudia Schneider-Danwitz; *Praxis fur Dialyse und Nierenkrankheiten - Arztezentrum Helle Mitte*: Wolfgang Seeger, Wolf-Adam Seeger, Britta Zemann; *Klinikum Bielefeld*: Christoph Stellbrink, Kristin Marx, Ekaterina Stellbrink, Britta Brettschneider, Stephanie Watson, Marion Iselt; *Studienzentrum Aschaffenburg*: Gerhard Klausmann, Inga-Nadine Kummer, Auguste Kutschat, Simone Streitenberger; *Universitatsklinikum Halle*: Matthias Girndt, Silke Markau, Ina Girakossyan, Claudia Hanf; *Klinikum St. Georg Leipzig*: Joachim Beige, Ralph Wendt, Ulrike Schmidt; *Studienzentrum Nephrologie Nurnberg-Langwasser*: Andreas Schneider, Roland Veelken, Claudia Donhauser, Auguste Kutschat; *UBAG fur Nephrologie und Dialyse Neckarsulm*: Luis Becker, Nexhat Miftari, Ricarda Wolfling, Sarah Morlok; *Universitatsklinikum Dresden*: Christian Hugo, Alexander Paliege, Jens Passauer, Julian Stumpf, Annegret Fleischer, Kerstin Haaser; *Universitatsklinikum Mannheim*: Bernhard Kraemer, Jan Jochims, Bernd Kruger, Claudia Foellinger, Anastassiya Reisler; *Nierenzentrum Wiesbaden*: Frank Strutz, Stefan Haack, Ursula Hohenstatt; *Universitatsklinikum Jena*: Martin Busch, Konstantin Herfurth, Gunter Wolf, Rainer Paul; *Studienzentrum fur Nieren- und Hochdruckerkrankungen Hannover*: Hermann Haller, Jessica Kaufeld, Jan Menne, Elisabeth Bahlmann-Kroll, Angela Bergner; *Universitatsklinikum Augsburg*: Horst Weihprecht, Aydin Er, Florian Sonntag, Elif Turan, Michael Wittmann, Franziska Klauser, Eva Voigt; *Nephrologisches Zentrum Gottingen*: Volker Schettler, Egbert Schulz, Madlen Rohnstock, Elke Schettler; *Universitaetsklinik Ulm*: Bernd Schroppel, Rene van Erp, Martin Kachele, Ulla Ludwig, Lena Schulte-Kemna, Waltraud Kmietschak, Elke Preiss, Martina Ruocco; *AGAPLESION Markus-Krankenhaus*: Gunnar Heine, Martin Brzoska, Sebastian Gabel, Christina Büttner, Asma Sabarai; *Universitatsklinikum Regensburg*: Bernhard Banas, Tobias Bergler, Yvonne Ehrl, Franz Putz, Antonia Schuster, Stefanie Kuhn, Torsten Schramm; *DaVita Viersen - Nettetal*: Stefan Degenhardt, Gerhard Schmidt, Lea Weiland, Ulrike Giebeln-Hudnell; *Klinikum Braunschweig*: Jan Kielstein, Gabriele Eden, Brigitte Fuchs, Gina Morig, Manuela Winkler; *Nephrocare Mettmann*: Michael Koch, Sendogan Aker, Annika Küpper, Manuela Martin; *Vivantes Klinikum Neukolln*: Harald Darius, Charalampos Kriatselis, Carl-Philipp Roesch; Astrid Maselli, *Robert-Bosch-Krankenhaus Stuttgart*: Dominik Alscher, Markus Ketteler, Moritz Schanz, Severin Schricker, Bianka Rettenmaier, Andrea Schwab

United States: *Clinical Advancement Center*: Pablo Pergola, Irene Leal, Melissa Cagle, Anna Romo, Anthony Torres; *Seacoast Kidney and Hypertension Specialists*: Sucharit Joshi, Kulli Barrett, Alexis Africano, Vicki Dodds, Dorleena Gowen, Ashlee Morris; *Total Research Group, LLC*: Juan Fernandez, Guillermo Jimenez, Ricardo Viera, Kendaling Bruce, Ryan Barrios, Maylin Garcia, Kerelyn Garcia, Iradis Leal; *Nephrology Consultants, LLC*: David Tietjen, David Bains, Carlo Castillo, Genielle Brewer, Justin Davis, Natalie Freking, Brittany Golson, Sally Ham, Jesslyn Roesch; *Sumter Medical Specialists*: Pusadee Suchinda, Shameem Beigh, Usah Lilavivat, Joyce Bilton, Kim Bocchicchia; *Yale University*: Jeffrey Turner, Neera Dahl, Aldo Peixoto, Yasemin Kavak, Lauren Liberti, Hari Nair, Nicolas Page, Stephanie Rosenberg, Kathryn Simmons; *Northwestern University*: Tamara Isakova, Rebecca Frazier, Rupal Mehta, Anand Srivastava, Patrick Fox, Jonathan Hecktman, Alexander Hodakowski, Carlos Martinez, Rachel Phillips, Alexis Stevenson; *University of Kansas Medical Center*: Reem Mustafa, Kyle Jansson, Cassandra Kimber, Jason Stubbs, Ahmad Tuffaha, Sri Yarlagadda, Debbie Griffin, Elisabeth Laundy, Zhuo Tang; *Providence Sacred Heart Medical Center and Childrens Hospital*: Radica Alicic, Katherine R. Tuttle, Ann Cooper, Lisa Davis; *East Coast Research Institute*: Ashwini Gore, Rebecca Goldfaden,

Leslie Harvill, Lisa Hichkad, Barry Johns, Thomas Jones, Kayla Merritt, Jennifer Sheldon, Jennifer Stanfield, Lindsay Alexander, Kaitlyn Preston, Lindsey Wood; *Monument Health*: Rajesh Pradhan, Roger DeRaad, Kelli McIntosh, Louis Raymond, Michael Shepperd, Susan McLaughlin, Mary Seifert, Andrew Shepherd; *Mountain Kidney & Hypertension Associates*: Joseph Aiello, William Durham, Laurie Loudermilk, John Manley, Sabrina Burnette, Stephanie Evans, Tara Johnson; *Texas Institute for Kidney and Endocrine Disorders*: Lance Sloan, Judy Ann Acosta, Stacy Gillham, Katia Sloan, SueAnn Squyres; *Wake Forest University Health Sciences*: Michael Rocco, Amret Hawfield, Ben Bagwell, Lauren Richmond; *Chase Medical Research*: Joseph Soufer, Subha Clarke, Amanda Aliu, Kristine Calabrese, Amanda Davis, Veronica Poma, Tracy Spinola; *East Coast Institute for Research LLC*: James Magee, Ricardo Silva, Rushab Choksi, Lorraine Dajani, John Evans, Anil George, Rebecca Goldfaden, Prasanth Krish, Gerard Martins, Mae Sheikh-Ali, David Sutton, Freda Driver, Abraham Hanburry, Laura Hume, Amber Hurst, Matthew Taddeo, Marla Turner, Veronica Yousif; *University of Utah Health Sciences*: Srinivasan Beddhu, Laith Al-Rabadi, Nikita Abraham, Amalia Caamano, Judy Carle, Victoria Gonce, Kaitlyn Staylor, Na Zhou; *University of Texas Health Science Center at San Antonio*: Shweta Bansal, Manoj Bhattarai, Kumar Sharma, Subrata Debnath, Aliseiya Garza, Chakradhar Velagapudi; *Academy of Diabetes, Thyroid, and Endocrine, PA*: Sergio Rovner, Javier Almeida, Pablo Casares, Verlaine Stewart-Ray, Rene Almaraz, Renata Dayrell, Ana Moncada, Ricardo Pulido, Roxana Rodriquez; *East Coast Institute for Research*: James Magee, Wasim Deeb, Kathryn DeGoursey, Rodel Gloria, Trevor Greene, Robert Miller, Edward Pereira, Miguel Roura, Mae Sheikh-Ali, David Sutton, Debbie Domingo, Sasha Dorestin, William Hodge, Cathy Jackson, Deborah Lund, Katrina Taylor; *Aventiv Research*: Kenneth Boren, Brittany Cleveland, Sandra Gaiser, Mandeep Sahani, Logan Aldrich, Exodus Edmerson, Edmond Limon, Cole Valletta, Patricia Vasquez; *St. Clair Nephrology Research*: Christopher Provenzano, Navkiranjot Brar, Heather Henderson, Bellovich Keith, Qur Khai, Quresh Khairullah, Gail Makos, Joel Topf, Sherry Gasko, Rosemarie Henschel, Kaitlin Knapp, Teresa Kozlowski, Paula LaFleur, Ashwathy Varughese; *Kaiser Permanente San Diego*: Hui Xue, Patricia Wu, Olga Arechiga, Shan Darbeau, Michael Fechter, Stephanie Martinez; *Hanson Clinical Research Center*: Lenita Hanson, Nyla Cooper, Arelis Madera, Jay Cadorna, Rita Sheridan, Helen Sparks; *Saint Elizabeth Healthcare*: Bradley Eilerman, Susanne Bodine, Wael Eid, Rebecca Flora, Amber Avery, Cashmere Hardy; *Thomas Jefferson/ARIA Health Northeast Endocrine Metabolic Associates*: Mihaela Biscoveanu, Steven Nagelberg, Tracey Cummins; *Emory University*: Frederic Rahbari-Oskoui, Anju Oommen, Zohreh Forghani, Stacie Hitchcock, Darya Hosein, Diane Watkins; *East Coast Institute Research, LLC*: Minesh Patel, Anthony Lambert, Elizabeth Newman, Autumn Wood, Tammy Ross, Stephany Topping; *Kidney Care and Transplant Services of New England*: Jeffrey Mulhern, Lorna Murphy, Ann Vasseur; *Brookview Hills Research Associates LLC*: Gregory Greenwood, Alexander Hadley, Denise Laurienti, Christopher Marshall, Nicholas McLean, Scott Satko, Brandy Caudill, Jacob Maris, Janice Rogers, Cindy Vanhoy; *Cleveland Clinic*: George Thomas, Georges Nakhoul, John O'Toole, Jonathan Taliercio, Leslie Cooperman, Marina Markovic, Barbara Tucky; *Salem V.A. Medical Center*: Devasmita Dev, Alia Hasan, Hima Yalamanchili, Namita Jain, Lesley McNeil, Eric Wines; *Medstar Health Research Institute*: Jean Park, Adline Ghazi, Mia Hamm, Tejas Patel; *University of North Carolina Hospital*: Amy Mottl, Emily Chang, Vimal Derebail, Emmie Cole, Anne Froment, Sara Kelley, Jordan Osmond Foster; *Olive View - UCLA Medical Center*: Vahid Mahabadi, Golriz Jafari, Anita Kamarzarian, Wendy Arriaga, Daisy Arteaga, Rosario Machicado, Genesis Naverrete; *P&I Clinical Research, LLC*: Prashant Kumar, Imran Nazeer, Karina Urquia, Tammi Glider, Vickie Jones, Savannah Rucker, Jennifer Wiley; *Pioneer Research Solutions*: Rahul Pandey, Jesus Arroyo, Harish Pariani, Mohammad Ahmad, Shahin Mozaffari, Erika Perez; *Los Angeles Biomedical Research Institute at Harbor-UCLA Medical Center*: Matthew Budoff, Sion Roy, Divya Birudaraju, Ahmed Ghanem, Sajad Hamal; *Research Institute of Dallas*: Stephen Aronoff, Elisa Joye Petr, Richard Sachson, Jaime Wiebel, Sana Akram, Laurie Jones, Curtis Knight; Maurie Tarlac; *Renal Disease Research Institute*: Shahbaz Ahmed, Harold Szerlip, Akinwande Akinfolarin,

Ankit Mehta, Shana Camp, Cindy Castro, Zanaida Cooper, Jessica Terry; *Clinical Research Consultants*: Ahmed Awad, Bhavya Kothapalli, Ryan Lustig, Serine Alfaress, Hyder Jasim, Mary Parrigon; *Lexington V.A. Health Care System*: Dennis Karounos, Sadiq Ahmed, Maggie Berry, Ruth Oremus; *VA Southern Nevada Healthcare System*: Carlos Hernandez-Cassis, Elias Ugwu, Nazia Junejo, Nancy Suazo; *University of Florida Health*: Mark Segal, Amir Kazory, Sherry Brown, Tristan Daniels, Sofia Dayi, Renee Hogan, Kathy McCray, Jennifer Stickley; *University Hospitals Cleveland Medical Center*: Mahboob Rahman, Mirela Dobre, Lavinia Negrea, Aparna Padiyar, Nishigandha Pradhan, Arash Rashidi, Nagaraju Sarabu, Vicki Donley, Tricia Young; *Midland Florida Clinical Research Center*: Godson Oguchi, Judepatricks Onyema, Kahla Damianik, Jack Dienes, Judith Plummer-Morgan, Marilyn Roman, Mauver Skipper, Stacey-Ann Villaruel, Krystle Williams; *Cedar Crosse Research Center*: Danny Sugimoto, Jeffrey Dugas, Ismeal Ahmed, Jamie Bhairoo, Dolores Rijos, Huzaifa Salim

UK: *Oxford University Hospitals*: Richard Haynes, William G. Herrington, Doreen Zhu, Madita Gavrila, Kathryn Lafferty, Ria Rabara, Sally Ruse, Maria Weetman; *Southmead Hospital, Bristol*: James Bushnell, Albert Power, Alison Jenkins, Stefanie Jones, Amanda Scott; *Nottingham City Hospital*: Cath Byrne, Mark Jesky, Alison Cowley, Emma McHaffie, Holly Waterfall; *Dorset County Hospital*: Jo Taylor, Laura Bough, Thomas Phillips, Barbara Winter-Goodwin; *King's College Hospital, London*: Sui Phin Kon, Iain MacDougall, Eirini Lioudaki, Sapna Shah, Claire Sharpe, Francisco Aguilar, Abegail Hernandez Pena, Conception Pugay, Amelia Te; *Queen Elizabeth Hospital Birmingham*: Hugh Finn, Wasim Hanif, Samiul Mostafa, Alice Aitken, Katharine Draxlbauer, Evelina Grobovaite, Jennifer Kearney, Theresa McCarthy; *Royal Cornwall Hospital*: Giorgio Gentile, Duncan Browne, Palanichamy Chellamuthu, Tabinda Dugal, Terri Chant, Laura Jones, Emily Laity, Megan Miners, James Muir, Elizabeth Swanson; *Imperial College Healthcare NHS Trust*: Andrew Frankel, James Tomlinson, Marlon Alegata, Rashid Almasarwah, Anthoula Apostolidi, Maria Vourvou, Thomas Walters; *Royal Derby Hospital*: Maarten Taal, Hari Dukka, Nitin Kolhe, Carly McDonald, Kelly White; *The Queen Elizabeth Hospital, King's Lynn*: Shiva Ugni, Smita Gunda, Rotimi Oluyombo, Vicki Brindle, Ping Coutts, Tracy Fuller, Evelyn Nadar; *Princess Royal Hospital, Telford*: Suresh Ramadoss, Denise Donaldson, Nichola Motherwell, Susannah Pajak, Louise Tonks; *Hull Royal Infirmary*: Sunil Bhandari, Richard Bodington, Adil Hazara, Dominic Fellowes; *University Hospital Aintree*: Christopher Wong, Christopher Goldsmith, Sherald Barnes, Ann Bennett, Claire Burston, Samantha Hope, Nicola Hunt, Lini Kurian; *UHNM Royal Stoke University Hospital*: Richard Fish, Daniela Farrugia, Judy Lee, Emma Sadler, Hannah Turner; *Belfast City Hospital*: Christopher Hill, Henry Brown, Agnes Masengu, Peter Maxwell, Nina Bleakley, Hugh Murtagh; *West Suffolk NHS Foundation Trust*: William Petchey, Vivian Yiu, Joanne Kellett, Angharad Williams; *Royal Devon and Exeter Hospital*: Helen Clarke, Victoria Carnall, Sarah Benyon, Caroline Blake, Stephanie Estcourt, Jane Piper; *Daisy Hill Hospital*: Neal Morgan, Carolyn Hutchinson, Teresa McKinley; *Ulster Hospital, Dundonald*: Alastair Woodman, Judi Graham, Niall Leonard, John Smyth, Vicki Adell, Samantha Hagan; *Royal Free London NHS Foundation Trust*: Ben Caplin, Amin Oomatia, Eleanor Damian, Toluleyi Sobande; *Kent & Canterbury Hospital*: Tim Doulton, Michael Delaney, Mahmoud Montasser, Jenny Hansen, David Loader, Angela Moon, Frances Morris; *Salford Royal NHS Foundation Trust*: Smeeta Sinha, Chukwuma Chukwu, Amy Hudson, Diane Campbell, Melanie Kershaw, Stephanie Whittaker; *Brighton and Sussex University Hospital's NHS Trust*: Ayesha Irtiza-Ali, Farid Ghalli, Heba Nosseir, Allison Leslie, Kate Trivedi; *University Hospital of Wales, Cardiff*: Donald Fraser, Mohammad Alhadj Ali, Sian Griffin, Farah Latif, Justyna Witczak, Alexa Wonnacott, Lynda Jeffers, Yvette Webley; *Edinburgh Royal Infirmary*: Paul Phelan, Eve Miller-Hodges, Ailsa Geddes, Margaret Glenwright, Amy Hunter; *Gloucestershire Hospitals NHS Foundation Trust*: Thomas Pickett, Jim Moriarty, Linda Hill, Amanda Tyler; *University Hospitals Coventry and Warwickshire*: Waqar Ayub, Gail Evans, Sue Hewins, Davina Hewitt, Kerry Read; *Ninewells Hospital*: Samira Bell, Leanne Cosgrove, Rachel Craik, Shona Murray; *Royal Berkshire Hospital, Reading*: Nitin Bhandary, Holly Coles, Rashmi Easow, Maya

Joseph; *Northern General Hospital, Sheffield*: Arif Khwaja, Yvonne Jackson, Angeline Mbuyisa, Rachel Sellars; *Darent Valley Hospital, Dartford*: Nihil Chitalia, Cynthia Mohandas, Anca Gherman, Charlotte Kamundi, Olumide Olufuwa; *Royal London Hospital*: Kieran McCafferty, Adedolapo Adeleke, Cara Healy, Damini Jeyarajah, Edward Kinsella-Perks; *Ipswich Hospital*: Richard Smith, Brian Camilleri, Carol Buckman, Jenny Finch, Vanessa Rivers; *University Hospitals Plymouth NHS Trust*: Andrew Connor, Sheila Carr, Lisa Shainberg; *Cheltenham General Hospital*: Thomas Pickett, Linda Hill, Amanda Tyler; *St. James's University Hospital, Leeds*: Andrew Lewington, Richard Baker, Suzannah Dorey, Kay Tobin, Rosalyn Wheatley; *St. George's University Hospitals NHS Foundation Trust*: Debasish Banerjee, Richard Hull, Sharirose Abat, Riny Paul; *Norfolk and Norwich University Hospitals*: Mahzuz Karim, Zay Htet, Rotimi Oluyombo, Saad Tufail, Ravi Varma, Karen Convery, Deirdre Fottrell-Gould, Lisa Hudig, Emily Tropman; *Walsall Healthcare NHS Trust*: Thahir Abdul-Samad, Anne Grace, Marie Phipps; *St Helier Hospital, Carshalton*: Rebecca Suckling, Subash Somalanka, Bhrigu Sood, Pauline Swift, Sarah Acheampong, Kwame Ansu, Martia Augustin; *Wessex Kidney Centre, Queen Alexandra Hospital, Portsmouth*: Anna Sampson, Lynn Vinall, Kim Wren; *St Bartholomew's Medical Centre*: Shamila Wanninayake, Nicholas Wooding, Heather Edwards, Lydia Owen; *Antrim Area Hospital*: Stephanie Bolton, Marion Carson, Michael Matthews; *University Hospitals of Leicester*: Nigel Brunskill, Jorge Jesus-Silva, Alex Howson, Mary Quashie-Akponeware; *North Middlesex University Trust Diabetes Department, North Middlesex University Hospital*: Hilary Tindall, Chidambaram Nethaji, Helen Eldon; *Glasgow Clinical Research Facility, Queen Elizabeth University Hospital*: Rajan Patel, Patrick Mark, Alastair Rankin, Michael Sullivan, Kirsty Forsyth, Rowan McDougall; *Great Western Hospital, Swindon*: Tanaji Dasgupta, Louisa Davies, Maggie Ryder; *Hathaway Medical Centre, Chippenham*: Philip Grimmer, Clare Macdonald, Mary Webster; Newcastle: Timothy Ellam, Edwin Wong, Christine Meshykhi, Andrea Webster, Peter Wilson; *Lister Hospital*: Enric Vilar, Jocelyn Berdeprado, Eunice Doctolero, Lily Wilkinson; *Altnagelvin Hospital, Western Health & Social Care Trust*: Frank McCarroll, Hesham Ammar, Ying Kuan, Conor Moran, Girish Shivashankar, Ryan Campbell, Deborah Glowski, Paula McDermott; *Oakenhurst Medical Practice, Blackburn*: Amar Ali, Zuber Patel, Christine Bond, Gillian Whalley

China: *National Clinical Research Center of Kidney Diseases, Jinling Hospital, Nanjing University School of Medicine*: Haitao Zhang, Peiling Chen, Yu An, Yinghua Chen, Liu Yang, Lihua Zhang, Tingting Kan, Ling Zhu; *The Second Affiliated Hospital of Army Medical University, PLA*: Jinghong Zhao, Weiping Hou, Jing Wu; *Beijing Anzhen Hospital, Capital Medical University*: Hong Cheng, Weijing Bian, Zhirui Zhao; *Henan Provincial People's Hospital*: Fengmin Shao, Huixia Cao, Xiaojing Jiao, Peiyuan Niu; *Shanghai Fifth People's Hospital, Fudan University*: Jianying Niu, Yu Chen, Lihong Zhang; *Huazhong University of Science and Technology Union Shenzhen Hospital*: Shenglang Zhu, Haiyan Lin, Shaopeng Yao, Jiehui Chen, Ying Jiang; *The second affiliated hospital of Zhejiang University School of Medicine*: Ying Hu, Huaying Xiao, Fuye Yang; *Shenzhen People's Hospital*: Xinzhou Zhang, Baochun Guo, Qiu Jin, Lixia Liu; *Xiangya Hospital, Central South University*: Xiangcheng Xiao, Yanyun Xie, Ting Meng; *Wuhan Fourth Hospital*: Chuanwen Xu, Jie Huang, Yanmei Xu; *Suzhou Kowloon Hospital*: Weixin Kong, Xiaoliang Wang, Qianpan Liu,; *Jinzhou Central Hospital*: Xueying Wang, Ming Gao; *Zhuzhou Central Hospital (Nephrology)*: Xiumei Hu, Ying Lu; *Sichuan Provincial People's Hospital*: Li Wang, Kun Peng, Wei Wang; *Fuwai Hospital, Chinese Academy of Medical Sciences*: Qiuhong Gong, Jianfang Cai, Xiaojue Li, Xuejiao Liu, Haitao Zhang, Shuhan Zhou; *Zhuzhou Central Hospital (Endocrinology)*: Hong Liu, Yao Weng, Shuai Tang, Yao Yao; *The Central Hospital of Wuhan*: Shi Zhao, Chen Cheng, Wei Wei, Na Li

Malaysia: *Hospital Kajang*: Sadanah Aqashiah Mazlan, Alia Zubaidah Bahtar, Elliyyin Katiman, Noraini Othman; *Hospital Tuanku Ja'afar*: Lily Mushahar, Nurdiana Mazlan, Nur Sharafina Safiee, Sarasa Ramasamy; *Hospital Selayang*: Hin Seng Wong, Hajar Ahmad Rosdi, Esther Zhao Zhi Tan, Ju Fan Tay; *Hospital Taiping*: Kok Seng Teng, Hasnah

Yahaya; *Hospital Sultanah Aminah*: Wen Jiun Liu, Lik Wee Ee, Kenneth Kay Leong Khoo, Yuana Mohd Yusoff; *Hospital Tengku Ampuan Afzan*: Fariz Safhan Mohamad Nor, Mohd Kamil Ahmad, Mohd Ramli Seman; *Hospital Umum Sarawak*: Clare Hui Hong Tan, Laura Lui Sian Ngu, Jaime Yoke May Chan, Javelin Peji; *Hospital Raja Permaisuri Bainun*: Chek Loong Loh, Yee Yan Lee, Sridhar Ramanaidu, Kah Mean Thong, Yik Hong Wong, Suria Junus; *Hospital Sultanah Bahiyah*: Chen Hua Ching, Mohammad Faisal Asmee, Ku Ruziana Ku Md Razi, Chun Leong Low, Christopher Sze Bing Sim, Zhang Duan Tham, Noor Kamila Abdullah; *Hospital Sultan Abdul Halim*: Tai Meng Chen, Yong Chieh Chan, Eason Chang, Huan Yean Kang, Kai Quan Lee, Sue Ann Lee, Aik Kheng Lee, Jeevika Vinathan; *Universiti Kebangsaan Malaysia Medical Centre*: Rizna Abdul Cader, Ruslinda Mustafar, Lydia Kamaruzaman, Rozita Mohd, Rahimah Ismail; *Hospital Kulim*: Chong Men Leong, Chee Koon Low, Liang Wei Wong, Norlezah Adnan, Sabariah Ibrahim; *Hospital Kuala Lumpur*: Mohamad Zaimi Abdul Wahab, Sunita Bavanandan, Yik Shen Lim, Zhang Duan Tham, Wan Hazlina Wan Mohamad, Siti Munirah Jaafar, Nur Ashykeen Mohd Fauzi, Aziee Sudin; *University Malaya Medical Centre*: Soo Kun Lim, Chye Chung Gan, Albert Hing, Wan Ahmad Faizal Alaidin Razali; *Hospital Pulau Pinang*: Yew Fong Liew, Chelsia Bao Tyng Chan, Mei Chih Cheng, Yu Chen Ong, Loke Meng Ong, Farah Amalina Mohamed Affandi; *Hospital Melaka*: Korina Rahmat, Ban Chai Peng, Masayu Amat; *Hospital Pakar Sultanah Fatimah*: Nuzaimin Hadafi Ahmad, Doo Yee Mah, Yi Loon Tye, Zaid Azhari, Siti Nabilah Mohamad Zaini, Mohd Aidil Musa; *Hospital Ampang*: Norazinizah Ahmad Miswan, Rafizanur Ramli, Nor Aziah Ahmad; *Hospital Serdang*: Bak Leong Goh, Nurul Izah Ahmad, Fairol Huda Ibrahim, Tze Jian Ng, Malini Shanmuganathan, Li Lian Tay; *Hospital Sultanah Nur Zahirah*: Zaiha Harun, Salmi Ramli, Nurul 'Ain Yusof, Rossenizal Abd Rahman; *Pusat Perubatan UiTM*: Muhammad Iqbal Abdul Hafidz, Nur Hidayati Mohd Sharif, Irda Yasmoon Awang

Japan: *Chubu Rosai Hospital*: Eitaro Nakashima, Rui Imamine, Makiko Minatoguchi, Yukari Miura, Miduki Nakaoka, Yoshiki Suzuki, Hitomi Yoshikawa; *Shin Clinic*: Koki Shin, Kanae Fujita, Misuzu Iwasa, Haruka Sasajima, Airi Sato; *Kansai Electric Power Hospital*: Yoshiyuki Hamamoto, Yuki Fujita, Takuya Haraguchi, Takanori Hyo, Kiyohiro Izumi, Toshiyuki Komiya, Sodai Kubota, Takeshi Kurose, Hitoshi Kuwata, Susumu Nakatani, Kaori Oishi, Saki Okamoto, Kaori Okamura, Jun Takeoka, Nagaaki Tanaka, Katsuya Tanigaki, Naohiro Toda, Koin Watanabe, Hiromi Komori, Rika Kumuji, Asako Takesada, Aya Tanaka; *Nagoya University Hospital*: Shoichi Maruyama, Tomonori Hasegawa, Akiko Ishiguro, Takuji Ishimoto, Kazuhiro Ito, Yutaka Kamimura, Noritoshi Kato, Sawako Kato, Hiroshi Kojima, Tomoki Kosugi, Kayaho Maeda, Masasi Mizuno, Shoji Saito, Hitomi Sato, Yuka Sato, Yasuhiro Suzuki, Akihito Tanaka, Yoshinari Yasuda, Fujiko Hasegawa, Maiko Hayashi, Shizuka Higashi, Kaho Shimamura, Momoko Sumi, Kazuki Tajima, Chimaki Unekawa, Kana Wakayama, Yukiko Wakita; *Ota diabetes clinic*: Takatoshi Otani, Ayako Imai, Sayaka Kawashima, Eri Kogure, Tomoe Sato, Misato Takezawa, Shinya Yoshida; *Fukui Prefectural Hospital*: Hideo Araki, Yuko Katsuda, Masahiro Konishi, Takahiro Matsunaga, Masashi Oe, Kunihiro Ogane, Masato Sakai, Tomoko Takahashi, Takahiro Yamano, Takuya Yokoyama, Hitomi Ito, Masayo Katayama, Emi Kuroda; *Medical Corporation Seijinkai Ikeda Hospital*: Toru Ikeda, Takuma Kojo, Etsuo Yoshidome, Rieko Mizumachi, Akane Yamamoto, Narihisa Yamasaki, Yoshihiko Yamasaki; *Okayama University Hospital*: Jun Wada, Jun Eguchi, Chigusa Higuchi, Akihiro Katayama, Masaru Kinomura, Masashi Kitagawa, Shinji Kitamura, Satoshi Miyamoto, Hiroshi Morinaga, Atsuko Nakatsuka, Ichiro Nojima, Kenichi Shikata, Hitoshi Sugiyama, Katsuyuki Tanabe, Kenji Tsuji, Haruhito Uchida, Mayu Watanabe, Chie Hashimoto, Takahiro Kato, Sayaka Yamamoto; *Tokai University Hospital*: Takehiko Wada, Masafumi Fukagawa, Naoto Hamano, Masahiro Koizumi, Hirotaka Komaba, Yosuke Nakagawa, Michiyo Iwamoto; *Fukuoka University Hospital*: Kosuke Masutani, Akane Katanosaka, Mayu Kiyota, Hikari Uchi, Yuka Ueda, Sonoka Yamamoto; *Kawasaki Medical School Hospital*: Hajime Nagasu, Seiji Itano, Tsukasa Iwakura, Hiroyuki Kadoya, Eiichiro Kanda, Naoki Kashihara, Kengo Kidokoro, Megumi Kondo, Tamaki Sasaki, Minoru Satoh, Atsuyuki Tokuyama, Reina Umeno, Yoshihisa Wada, Toshiya Yamamoto, Yu Yamanouchi,

Masumi Abe, Yoko Inukai; *Kobe University Hospital*: Wataru Ogawa, Shunichiro Asahara, Hideki Fujii, Shunsuke Goto, Yushi Hirota, Tetsuya Hosooka, Keiji Kono, Shinichi Nishi, Yuko Okada, Kazuhiko Sakaguchi, Kenji Sugawara, Michiko Takahashi, Tomoko Takai, Yoshikazu Tamori, Kentaro Watanabe, Miyu Kitajima, Misaki Nishi, Junko Wada; *Aichi Medical University Hospital*: Yasuhiko Ito, Hideki Kamiya, Akimasa Asai, Nao Asai, Saeko Asano, Shogo Banno, Yohei Ejima, Hanako Hase, Tomohide Hayami, Tatsuhito Himeno, Takahiro Ishikawa, Mayumi Ito, Shiho Iwagaitsu, Rina Kasagi, Yoshiro Kato, Makoto Kato, Koichi Kato, Takayuki Katsuno, Miyuka Kawai, Hiroshi Kinashi, Masaki Kondo, Masako Koshino, Naoya Matsuoka, Yoshiaki Morishita, Mikio Motegi, Jiro Nakamura, Hiromi Shimoda, Hirokazu Sugiyama, Shin Tsunekawa, Makoto Yamaguchi, Kazuyo Takahashi; *Juntendo University Hospital*: Hirotaka Watada, Takashi Funayama, Yasuhiko Furukawa, Tomohito Gohda, Hiromasa Goto, Hideyoshi Kaga, Yasuhiko Kanaguchi, Akio Kanazawa, Kayo Kaneko, Toshiki Kano, Masao Kihara, Shogo Kimura, Takashi Kobayashi, Masayuki Maiguma, Yuko Makita, Satoshi Mano, Tomoya Mita, Takeshi Miyatsuka, Maki Murakoshi, Masahiro Muto, Masami Nakata, Junichiro Nakata, Yuya Nishida, Nao Nohara, Takeshi Ogihara, Daisuke Sato, Junko Sato, Hiroaki Sato, Yusuke Suzuki, Ruka Suzuki, Hitoshi Suzuki, Miyuki Takagi, Yoshifumi Tamura, Toyoyoshi Uchida, Seiji Ueda, Miki Asawa, Minako Miyaji, Eri Nagashima, Yoshie Shibata, Eri Yanagisawa; *The University of Tokyo School of Medicine/Toranomon Hospital*: Takashi Kadowaki, Toshimasa Yamauchi, Masaomi Nangaku, Yosuke Hirakawa, Hiroshi Nishi, Nobuhiro Shojima, Satoko Horikawa, Yukiko Nakayama, Naoko Yamada, Yuki Omori; *Maebashi Hirosegawa Clinic*: Shintaro Yano, Miyabi Ioka, Nahoko Kuwabara, Remi Nagano, Megumi Nozawa, Yumi Osawa; *Shiga University of Medical Science Hospital*: Hiroshi Maegawa, Shinji Kume, Shinichi Araki, Itsuko Miyazawa, Katsutaro Morino, Ikuko Kawai, Masumi Sobata, Motoko Takaoka; *Koukan Clinic*: Yasushi Iwaita, Takashi Udagawa, Ami Inamori, Aya Kawase, Aya Yamanaka; *University of Tsukuba Hospital*: Hitoshi Shimano, Akiko Fujita, Hitoshi Iwasaki, Hirayasu Kai, Yoshinori Osaki, Chie Saito, Motohiro Sekiya, Ryoya Tsunoda, Kunihiro Yamagata, Rikako Nakamura, Aiko Yamada; *Center Hospital of the National Center for Global Health and Medicine*: Mitsuru Ohsugi, Motoharu Awazawa, Ryotaro Bouchi, Shota Hashimoto, Makiko Hashimoto, Tomoko Hisatake, Noriko Ihana, Koko Ishizuka, Kazuo Izumi, Hiroshi Kajio, Michi Kobayashi, Noriko Kodani, Koji Maruyama, Michihiro Matsumoto, Maya Matsushita, Tomoka Nakamura, Takehiro Sugiyama, Akiyo Tanabe, Aiko Terakawa, Kojiro Ueki, Yuko Orimo, Takako Ozawa, Eriko Takahira; *AMC Nishi-Umeda Clinic*: Yoshimitsu Yamasaki, Masakazu Haneda, Tadahiro Tomita, Saori Akimoto, Akihiro Fujimoto, Kenji Ishihara, Chiho Murakami, Akiyo Nishiyama, Yukiko Toyonaga, Kana Uozumi, Yukihiro Yamaji; *Jyoumou Ohashi Clinic*: Tetsuya Shigehara, Jun Okajyo, Yukihiro Shimizu; *Iwasaki internal medicine clinic*: Shingo Iwasaki, Yuki Fukao, Megumi Furusho, Shintaro Nunokawa; *Tohoku University Hospital*: Hideki Katagiri, Tomohito Izumi, Keizo Kaneko, Shinjiro Kodama, Mariko Miyazaki, Yuichiro Munakata, Tasuku Nagasawa, Yuji Oe, Hiroto Sugawara, Kei Takahashi, Kazushige Hirata, Keiko Inomata, Shoko Otomo, Taeko Uchida, Chigusa Yamashita; *Tokyo-eki Center-building Clinic*: Arihiro Kiyosue, Ryota Tamura

Canada: *CRIUCPQ*: Francois Dube, Marilene Bolduc, Marie-Christine Talbot; *University Health Network-Toronto General Hospital*: David Cherney, Leslie Cham, Vesta Lai, Josephine Tse; *Clinical Research Solutions Inc.*: Shivinder Jolly, Tabbatha Duck; *Interior Health Kelowna General Hospital*: Scott Lyle, Rachel Epp, Camille Galloway, Susan Haskett, Elizabeta Matvienko, Liam Paulsen; *London Health Sciences Centre*: Louise Moist, Kerri Gallo, Zabrina Lozon, Tina Ramsey, Brittany Whitmore; *St Paul's Hospital*: Adeera Levin, Bader Al-Zeer, Paula Macleod, Aoife O'Sullivan, Zainab Sheriff, Sam Tholl; *Cambridge Cardiac Care Centre*: Amritanshu Pandey, Samantha Armstrong, Bethelihem Gebeyehu, Patrick Toth; *LMC Clinical Research Inc. (Thornhill)*: Ronald Goldenberg, Mahsa Jahangiriesmaili, Shariff Sanguila, Neethi Suresh, Tanvi Talsania; *Vancouver General Hospital*: Nadia Zalunardo, Bader Al-Zeer, Paula Macleod, Aoife O'Sullivan, Zainab Sheriff; *CHU de Quebec-Universite Laval*: Mohsen Agharazii, Marie-Pier Roussel, Annie Saillant,

France Samson; *LMC Clinical Research Inc. Brampton*: Harpreet Bajaj, Miken Bhavsar, Parul Dhall, Gagandeep Dhillon, Bhupinder Grewal, Taniya Nimbkar; *CIUSSS Nord de l'ile de Montreal*: Francois Madore, Guylaine Marcotte; *LMC Clinical Research Inc. (Bayview)*: Oren Steen, Mathura Bullen, Shayani Raguwaran, Andre Valleteau; *CIUSSS de l'Estrie-CHUS, Hopital Fleurimont*: Marie-France Langlois, Christine Brown; *Lakeridge Health*: Andrew Steele, Melissa Garrity, Taneera Ghate, Holly Robinson, Michael Tolibas; *LMC Clinical Research Inc. (Ottawa)*: Chetna Tailor, Lauren Elliott, Christine McClary-Wright; *Fadia El Boreky Medicine Professional*: Fadia Boreky, Sameh Fikry, Ayesha Ali, Chintankumar Barot, Wagdy Basily, Bethelihem Gebeyehu, Thisun Saram, Vinay Varad; *LMC Clinical Research Inc (Etobicoke)*: Hasnain Khandwala, Alex Aguilera, Patricia Alvarez, Balwinder Gill, Nazihah Huda, Aamir Navivala, Daniel Pinto; *Kidney Care Centre-Fraser Health*: Micheli Bevilacqua, Elaine Fung, Geraldine Hernandez, Puneet Mann, Jaskiran Saini; *Institut de recherches cliniques de Montreal*: Remi Rabasa-Lhoret, Danijela Bovan, Marie Devaux

Italy: *Policlinico San Martino, Genova*: Roberto Pontremoli, Cecilia Barnini, Giovanna Leoncini, Luca Manco, Giulia Nobili; *Ospedale Casa Sollievo della Sofferenza, San Giovanni Rotondo*: Matteo Piemontese, Filippo Aucella, Rachele Grifa, Francesco Totaro; *Policlinico S. Orsola-Malpighi, Bologna*: Gaetano La Manna, Irene Capelli, Giuseppe Cianciolo, Sarah Lerario, Fulvia Zappulo; *Ospedale S. Giovanni di Dio, Firenze*: Alberto Rosati, Filippo Fani, Giuseppe Spatoliatore, Ester Baldini, Francesca Bianchini; *AOU Policlinico, Bari*: Loreto Gesualdo, Francesco Pesce, Maria Russo, Maria Zippo, Cesira Cafiero; *Ospedale Martini, Torino*: Daria Motta, Simona Bianco, Donatella Bilucaglia; *Ospedale Maggiore Policlinico, Milano*: Piergiorgio Messa, Laura Pavone, Federica Tripodi, Simone Vettoretti; *AOU Padova*: Paola Fioretto, Gianni Carraro, Filippo Farnia, Anna Postal; *Ospedale Sacro Cuore di Gesù, Gallipoli*: Alessandro D'Amelio, Antonio Cardone, Giovanni Piccinni, Annalisa Aloisi; *ASST Spedali Civili, Brescia*: Francesco Scolari, Federico Alberici, Alice Guerini, Chiara Saccà, Chiara Salviani, Roberta Zani; *AOU L. Vanvitelli, Napoli*: Luca De Nicola, Carlo Garofalo, Maria Elena Liberti, Roberto Minutolo, Luigi Pennino, Lucio Polese; AOU *Sant' Andrea, Roma*: Paolo Mené, Simona Barberi, Clorinda Falcone; *Ospedale Ignazio Veris delli Ponti, Scorrano*: Francesco Russo, Maurizio Caroppo; *Ospedale di Circolo, Desio*: Gennaro Santorelli, Rodolfo Rivera; AOU *Policlinico G. Martino, Messina*: Domenico Santoro, Alfio Giuffrida, Fortunata Zirino; *Ospedale Civile SS. Antonio e Biagio, Alessandria*: Cristina Calvi, Luca Estienne; *AOUI, Verona*: Giovanni Gambaro, Concetta Gangemi, Vittorio Ortalda, Giuseppina Pessolano; *Fondazione Policlinico Universitario Agostino Gemelli, Roma*: Giuseppe Grandaliano, Rocco Baccaro, Pietro Ferraro, Roberto Mangiacapra; *IRCCS Ospedale San Raffaele, Milano*: Marco Melandri, Nadia Foligno, Rita Quartagno, Giuseppe Vezzoli, Elena Brioni

Supplementary Tables & Figures

Supplementary Table 1: Characteristics of participants at baseline in those taking uric acid lowering therapy or colchicine at randomisation

	**Participants taking any uric-acid lowering therapy* or colchicine at baseline**		
	**Yes** N=2350	**No** N=4259	**Overall** N=6609
**DEMOGRAPHICS**			
Age at randomisation (years)			
Mean (SD)	63.9 (13.5)	63.8 (14.0)	63.8 (13.9)
Sex			
Male	1817 (77.3)	2600 (61.0)	4417 (66.8)
Female	533 (22.7)	1659 (39.0)	2192 (33.2)
Race (all regions)			
White	1344 (57.2)	2515 (59.1)	3859 (58.4)
Black	78 (3.3)	184 (4.3)	262 (4.0)
Asian	902 (38.4)	1491 (35.0)	2393 (36.2)
Mixed	9 (0.4)	12 (0.3)	21 (0.3)
Other	17 (0.7)	57 (1.3)	74 (1.1)
**PRIOR DISEASE**			
Prior diabetes†	971 (41.3)	2069 (48.6)	3040 (46.0)
History of cardiovascular disease‡	660 (28.1)	1105 (25.9)	1765 (26.7)
History of heart failure	274 (11.7)	384 (9.0)	658 (10.0)
History of gout	1171 (49.8)	536 (12.6)	1707 (25.8)
**CAUSE OF KIDNEY DISEASE**			
Diabetic kidney disease	574 (24.4)	1483 (34.8)	2057 (31.1)
Hypertension/renovascular	552 (23.5)	893 (21.0)	1445 (21.9)
Glomerular	685 (29.1)	984 (23.1)	1669 (25.3)
Other/unknown	539 (22.9)	899 (21.1)	1438 (21.8)
**CLINICAL MEASUREMENTS**			
Blood pressure (mmHg)			
Mean systolic (SD)	135.3 (17.8)	137.2 (18.5)	136.5 (18.3)
Mean diastolic (SD)	78.3 (11.6)	77.9 (11.9)	78.1 (11.8)
Body mass index (kg/m^2)			
Mean (SD)	30.0 (6.9)	29.6 (6.7)	29.7 (6.8)
**LABORATORY MEASUREMENTS**			
Estimated GFR (mL/min/1.73m^2)			
Mean (SD)	35.9 (13.0)	38.1 (15.1)	37.3 (14.4)
<30	874 (37.2)	1408 (33.1)	2282 (34.5)
≥30 <45	1046 (44.5)	1882 (44.2)	2928 (44.3)
≥45	430 (18.3)	969 (22.8)	1399 (21.2)
Urinary albumin-to-creatinine ratio (mg/g)			
Geometric mean (95% CI)	242 (224-262)	212 (200-225)	222 (212-233)
Median (Q1-Q3)	372 (61-1095)	310 (43-1056)	329 (49-1069)
<30	436 (18.6)	892 (20.9)	1328 (20.1)
≥30 ≤300	648 (27.6)	1216 (28.6)	1864 (28.2)
>300	1266 (53.9)	2151 (50.5)	3417 (51.7)
Serum uric acid (µmol/L)§			

Mean (SD)	378.7 (100.0)	459.6 (110.2)	431.3 (113.5)
NT-proBNP (ng/L)			
Geometric mean (95% CI)	170 (160-180)	170 (163-177)	170 (164-176)
Median (Q1-Q3)	159 (65-437)	161 (71-409)	160 (69-419)
**CONCOMITANT MEDICATION USE**			
RAS inhibitor	2047 (87.1)	3581 (84.1)	5628 (85.2)
Any diuretic therapy	1129 (48.0)	1686 (39.6)	2815 (42.6)
Loop diuretic	782 (33.3)	965 (22.7)	1747 (26.4)
Thiazide diuretic	416 (17.7)	706 (16.6)	1122 (17.0)
Lipid-lowering therapy	1529 (65.1)	2849 (66.9)	4378 (66.2)
Any uric acid lowering/gout therapy	2350 (100.0)	-	2350 (35.6)
Xanthine oxidase inhibitor[ǁ]	2215 (94.3)	-	2215 (33.5)
Primary uricosuric agent[¶]	118 (5.0)	-	118 (1.8)
Colchicine	111 (4.7)	-	111 (1.7)
**SMOKING & ALCOHOL**			
Ever smoked tobacco regularly[#]	1087 (46.3)	1862 (43.7)	2949 (44.6)
Ever drunk alcohol regularly[Δ]	1060 (45.1)	1614 (37.9)	2674 (40.5)
**5-YEAR RISK OF KIDNEY FAILURE** (KFRE, %), median (Q1-Q3)	11.1 (3.6-29.7)	8.5 (2.7-28.1)	9.4 (2.9-28.9)

Figures are n (%) or mean (SD) or median (Q1-Q3). *Includes xanthine oxidase inhibitors (allopurinol, febuxostat, topiroxostat); primary uricosuric agents (benzbromarone, probenecid); and colchicine. [†]Defined as participant-reported history of diabetes of any type, use of glucose-lowering medication or baseline HbA1c ≥48 mmol/mol at randomisation visit. [‡]Defined as participant-reported history of myocardial infarction, heart failure, stroke, transient ischaemic attack, or peripheral arterial disease. [§]To convert uric acid to mg/dL, divide by 59.48 (431.3 μmol/L ≈ 7.2 mg/dL). [ǁ]Allopurinol, febuxostat or topiroxostat. [¶]Benzbromarone or probenecid. [#]Defined as for most days for at least 1 year. [Δ]Defined as at least 1 day a week for at least 1 year. Abbreviations: GFR = glomerular filtration rate; ACR = albumin-to-creatinine ratio; RAS = renin-angiotensin system.

Supplementary Table 2: Characteristics of participants at baseline in those reporting a history of gout at randomisation

	Self-reported history of gout at randomisation		
	**Yes**<br>N=1707	**No**<br>N=4902	**Overall**<br>N=6609
**DEMOGRAPHICS**			
Age at randomisation (years)			
Mean (SD)	66.0 (12.1)	63.1 (14.3)	63.8 (13.9)
Sex			
Male	1329 (77.9)	3088 (63.0)	4417 (66.8)
Female	378 (22.1)	1814 (37.0)	2192 (33.2)
Race (all regions)			
White	1160 (68.0)	2699 (55.1)	3859 (58.4)
Black	82 (4.8)	180 (3.7)	262 (4.0)
Asian	436 (25.5)	1957 (39.9)	2393 (36.2)
Mixed	10 (0.6)	11 (0.2)	21 (0.3)
Other	19 (1.1)	55 (1.1)	74 (1.1)
**PRIOR DISEASE**			
Prior diabetes†	795 (46.6)	2245 (45.8)	3040 (46.0)
History of cardiovascular disease‡	535 (31.3)	1230 (25.1)	1765 (26.7)
History of heart failure	236 (13.8)	422 (8.6)	658 (10.0)
**CAUSE OF KIDNEY DISEASE**			
Diabetic kidney disease	485 (28.4)	1572 (32.1)	2057 (31.1)
Hypertension/renovascular	415 (24.3)	1030 (21.0)	1445 (21.9)
Glomerular	376 (22.0)	1293 (26.4)	1669 (25.3)
Other/unknown	431 (25.2)	1007 (20.5)	1438 (21.8)
**CLINICAL MEASUREMENTS**			
Blood pressure (mmHg)			
Mean systolic (SD)	136.5 (18.5)	136.5 (18.2)	136.5 (18.3)
Mean diastolic (SD)	77.4 (11.6)	78.3 (11.9)	78.1 (11.8)
Body mass index (kg/m^2)			
Mean (SD)	31.5 (7.0)	29.1 (6.6)	29.7 (6.8)
**LABORATORY MEASUREMENTS**			
Estimated GFR (mL/min/1.73m^2)			
Mean (SD)	34.4 (11.5)	38.3 (15.2)	37.3 (14.4)
<30	684 (40.1)	1598 (32.6)	2282 (34.5)
≥30 <45	775 (45.4)	2153 (43.9)	2928 (44.3)
≥45	248 (14.5)	1151 (23.5)	1399 (21.2)
Urinary albumin-to-creatinine ratio (mg/g)			
Geometric mean (95% CI)	188 (171-207)	235 (223-249)	222 (212-233)
Median (Q1-Q3)	275 (37-988)	346 (54-1094)	329 (49-1069)
<30	389 (22.8)	939 (19.2)	1328 (20.1)
≥30 ≤300	491 (28.8)	1373 (28.0)	1864 (28.2)
>300	827 (48.4)	2590 (52.8)	3417 (51.7)
Serum uric acid (µmol/L)§			
Mean (SD)	421.7 (121.3)	435.1 (110.2)	431.3 (113.5)
NT-proBNP (ng/L)			
Geometric mean (95% CI)	195 (182-209)	162 (155-168)	170 (164-176)
Median (Q1-Q3)	184 (78-491)	153 (67-396)	160 (69-419)
**CONCOMITANT MEDICATION USE**			

RAS inhibitor	1463 (85.7)	4165 (85.0)	5628 (85.2)
Any diuretic therapy	860 (50.4)	1955 (39.9)	2815 (42.6)
Loop diuretic	575 (33.7)	1172 (23.9)	1747 (26.4)
Thiazide diuretic	327 (19.2)	795 (16.2)	1122 (17.0)
Lipid-lowering therapy	1217 (71.3)	3161 (64.5)	4378 (66.2)
Any uric acid lowering/gout therapy	1171 (68.6)	1179 (24.1)	2350 (35.6)
Xanthine oxidase inhibitor[ǁ]	1125 (65.9)	1090 (22.2)	2215 (33.5)
Primary uricosuric agent[¶]	26 (1.5)	92 (1.9)	118 (1.8)
Colchicine	103 (6.0)	8 (0.2)	111 (1.7)
**SMOKING & ALCOHOL**			
Ever smoked tobacco regularly[#]	828 (48.5)	2121 (43.3)	2949 (44.6)
Ever drunk alcohol regularly[Δ]	852 (49.9)	1822 (37.2)	2674 (40.5)
**5-YEAR RISK OF KIDNEY FAILURE** (KFRE, %), median (Q1-Q3)	11.3 (3.9-30.6)	8.7 (2.7-28.0)	9.4 (2.9-28.9)

Figures are n (%) or mean (SD) or median (Q1-Q3). [†]Defined as participant-reported history of diabetes of any type, use of glucose-lowering medication or baseline HbA1c ≥48 mmol/mol at randomisation visit. [‡]Defined as participant-reported history of myocardial infarction, heart failure, stroke, transient ischaemic attack, or peripheral arterial disease. [§]To convert uric acid to mg/dL, divide by 59.48 (431.3 µmol/L ≈ 7.2 mg/dL). [ǁ]Allopurinol, febuxostat or topiroxostat. [¶]Benzbromarone or probenecid. [#]Defined as for most days for at least 1 year. [Δ]Defined as at least 1 day a week for at least 1 year. Abbreviations: GFR = glomerular filtration rate; ACR = albumin-to-creatinine ratio; RAS = renin-angiotensin system.

Supplementary Table 3: Proportion of the effect of empagliflozin on chronic eGFR slope explained by serum uric acid and other biomarkers at 2 months

Biomarkers	Absolute difference in chronic slope for empagliflozin vs placebo	Wald χ^2	% reduction in χ^2	Proportion of treatment effect explained (95% CI), %
None	1.36 (1.07, 1.66)	82.0	-	-
uACR	1.16 (0.87, 1.45)	61.0	25.6	15 (10,22)
Systolic blood pressure	1.28 (0.99, 1.58)	72.9	11.1	6 (3,11)
Diastolic blood pressure	1.35 (1.05, 1.64)	79.9	2.5	1 (0,4)
HbA1c	1.38 (1.08, 1.67)	83.3	-1.6	-1 (-3,1)
Uric acid	1.43 (1.13, 1.72)	88.7	-8.2	-4 (-9,-1)
uACR, systolic and diastolic blood pressure, HbA1c and uric acid	1.16 (0.87, 1.46)	61.3	25.2	15 (7,23)

Analyses restricted to 2586 participants with non-missing uric acid (and other biomarkers) at 2 months and a minimum of 2 eGFR measurements between 2 months and the final follow-up visit and therefore differ from previous publication (Staplin et al. Lancet Diabetes Endocrinol. 2024). Chronic slopes calculated for each individual participant using linear regression. The proportion of the treatment effect explained by 2-month biomarkers is estimated using the landmark method, adjusting a linear regression model with chronic slope as the dependent variable for 2 month values of biomarkers (categorised as approximate fifths). Analyses were additionally adjusted for baseline variables specified in the minimisation algorithm (age, sex, prior diabetes, eGFR, uACR and region). Confidence intervals for the proportion of treatment effect explained were constructed from bias-corrected and accelerated bootstrap intervals with 10000 replications. Replicating the analysis exploring proportion of treatment effect on the primary outcome and kidney disease progression outcome yielded consistent findings. Abbreviations: uACR = urinary albumin-to-creatinine ratio; HbA1c = glycated haemoglobin.

Supplementary Table 4: Summary of uric acid lowering effects in other large SGLT2 inhibitor trials

Trial	% with diabetes[1]	Mean baseline eGFR	Between group difference in uric acid, umol/L*	Time point at which between group difference in uric acid reported	Effects on gout events alone (where reported)
EMPA-REG OUTCOME[2]	100%	74	22.0	52 weeks	Time to first gout event: HR 0.81 (95% CI 0.56-1.16); 77 vs 47 events; 1.6 vs 2.0% (pooled empagliflozin vs placebo)
CANVAS[3]	100%	77	23.3	Study average (median follow-up 2.4 years); measured at every visit	Time to first gout event: HR 0.64 (95% CI 0.41-0.99); total 80 events; 2.0 vs 2.6% (canagliflozin vs placebo)<br>Recurrent gout events: HR 0.54 (95% CI 0.38-0.76); total 125 events; 2.8 vs 4.3%
CREDENCE[4]	100%	56	13.1	Study average (median follow-up 2.6 years); measured at every visit	-
DAPA-HF[5]	45%	66	50.0	12 months	-
EMPEROR-Reduced[6]	50%	62	66.6	4 weeks†	Time to first gout event: HR 0.70 (95% CI 0.45-1.08); 36 vs 49 events; 1.9 vs 2.6% (empagliflozin vs placebo)
VERTIS-CV[7]	100%	76	22.0	Study average (median follow-up 3.0 years); measured at every visit	-
DAPA-CKD	68%	43	Not reported		-
EMPEROR-Preserved[8]	49%	61	59.5	4 weeks	-
DELIVER	50%	61	Not reported		-
EMPA-KIDNEY	46%	37	25.6	Study average (median follow-up 2.0 years); measured at 2 and 18 months	*See Table 2*

*Where reported in mg/dL, approximately converted to µmol/L by multiplying by 59.48. †Difference persisted to week 100 but between group difference not quantified. eGFR = estimated glomerular filtration rate, HR = hazard ratio, CI = confidence interval.

---

[1] Characteristics for all presented trials including proportion with diabetes and mean eGFR at baseline are summarised in: Nuffield Department of Population Health & SGLT2 inhibitor Meta-Analysis Cardio-Renal Trialists' Consortium. 2022. Lancet.
[2] Ferreira JP et al. 2021. Diabetes Obes Metab.
[3] Li J et al. 2020. Kidney International. & Li J et al. 2019. Lancet Rheumatol.
[4] Doi Y et al. 2023. Diabetes Obes Metab.
[5] McDowell K et al. 2022. Eur J Heart Fail.
[6] Doehner W et al. 2022. Eur Heart J.
[7] Segar MW et al. 2022. Diabetes Obes Metab.
[8] Green J et al. 2023. Metabolism.

Supplementary Figure 1: Correlation between serum uric acid & estimated glomerular filtration rate at randomisation by sex

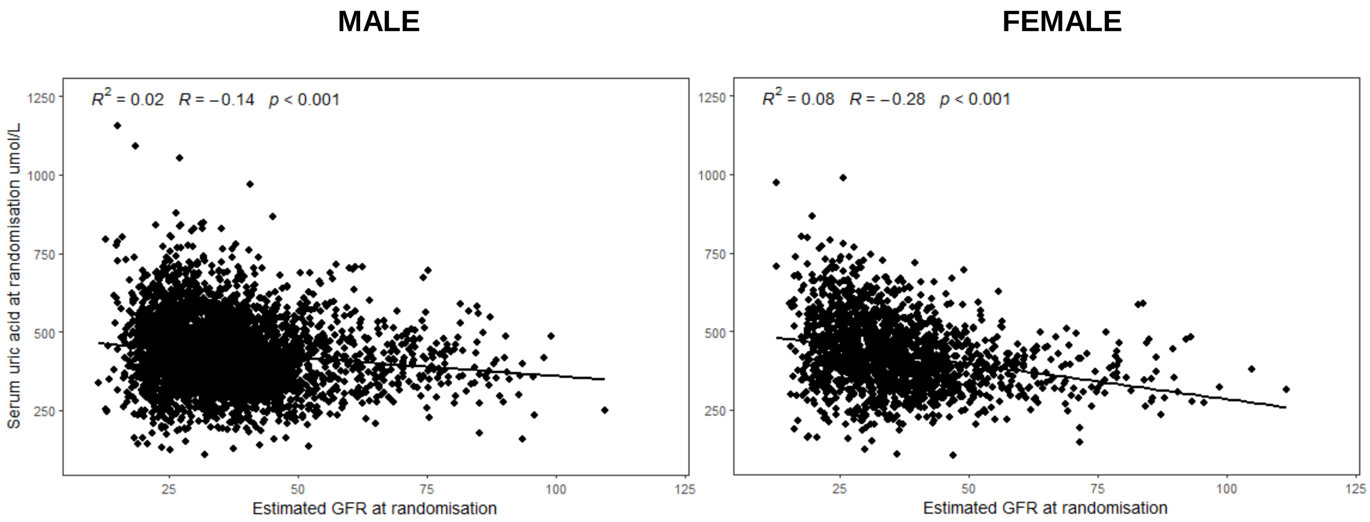

R = Spearman correlation coefficient. To convert uric acid to mg/dL, divide by 59.48.

Supplementary Figure 2: Relationship between baseline serum uric acid and first gout events in those allocated to placebo

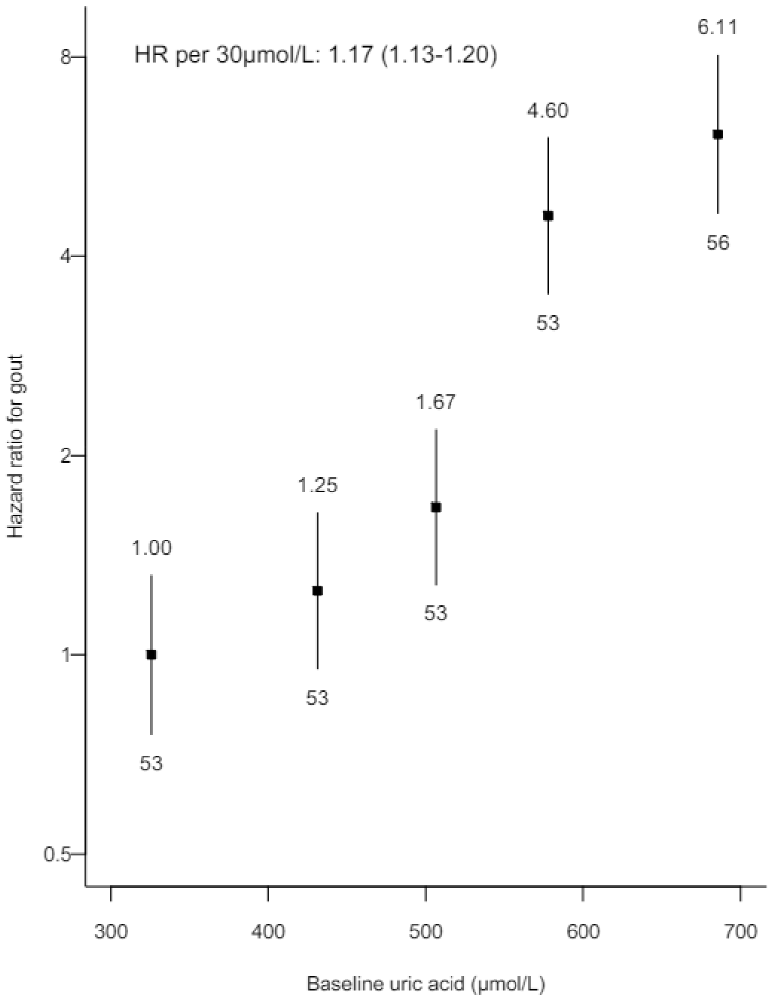

Hazard ratio for the time to first gout event in 2590 participants allocated to placebo who had a baseline measurement of serum uric acid (μmol/L). Baseline uric acid was categorised into fifths to ensure approximately equal numbers of gout events in each category. Cox regression model adjusted for the covariates used in the minimisation algorithm (categories of age, sex, diabetes, estimated glomerular filtration rate, urinary albumin-to-creatinine ratio and region). To convert uric acid to mg/dL, divide by 59.48 (500 μmol/L ≈ 8.4 mg/dL).

Supplementary Figure 3: Relationship between history of previous gout; uric acid lowering therapy or colchicine; and hyperuricaemia at randomisation

	Serum uric acid at randomisation (µmol/L)	
	**Mean±SD**	**N(%) ≥470***
**Overall**	431.3±113.5	1724 (33.4)
Male	433.0±114.1	1175 (34.0%)
Female	427.9±112.3	549 (32.1%)

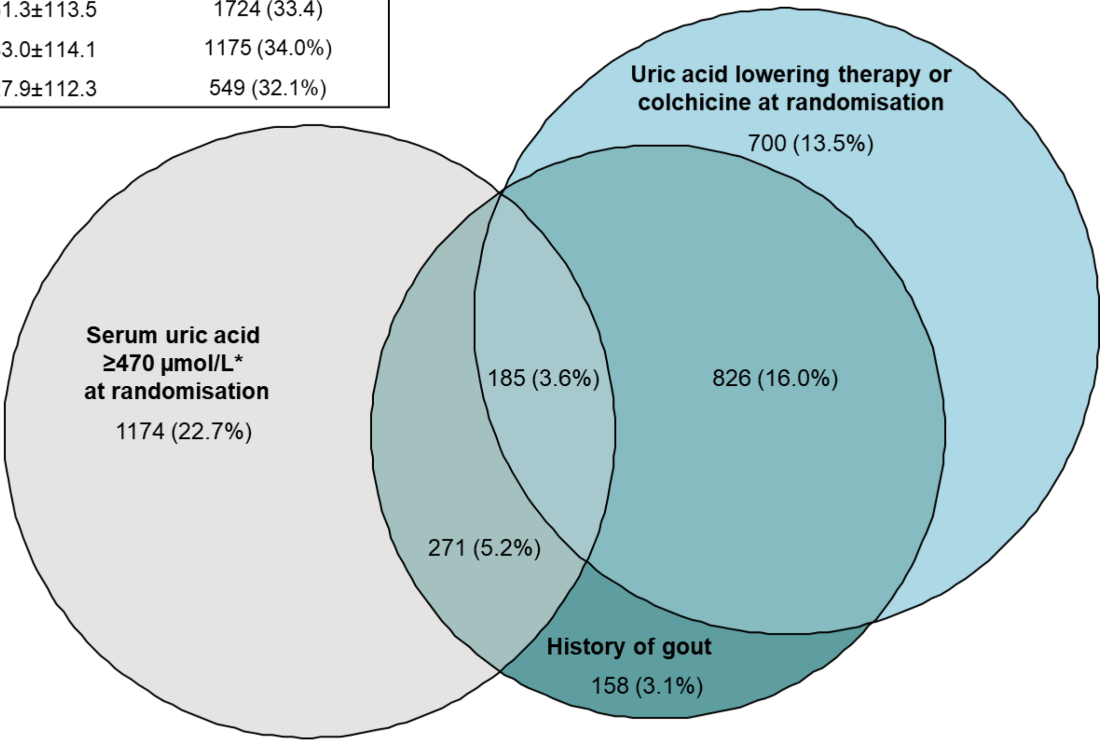

Participants (with baseline uric acid measurement) not fulfilling any of these criteria 1854 (35.9%)

Includes only participants with uric acid measured at randomisation, n=5168 (missing n=1441). Uric-acid lowering therapy includes xanthine oxidase inhibitors and primary uricosuric drugs (see Methods). *Corresponds to the top third of those with uric acid measured at randomisation, used in subgroup categorisation for time-to event analyses (Figure 3). To convert uric acid to mg/dL, divide by 59.48 (431 µmol/L ≈ 7.2 mg/dL; 470 µmol/L ≈ 7.9 mg/dL).

Supplementary Figure 4: Absolute and relative effects of empagliflozin versus placebo on serum uric acid in subgroups defined by baseline characteristics

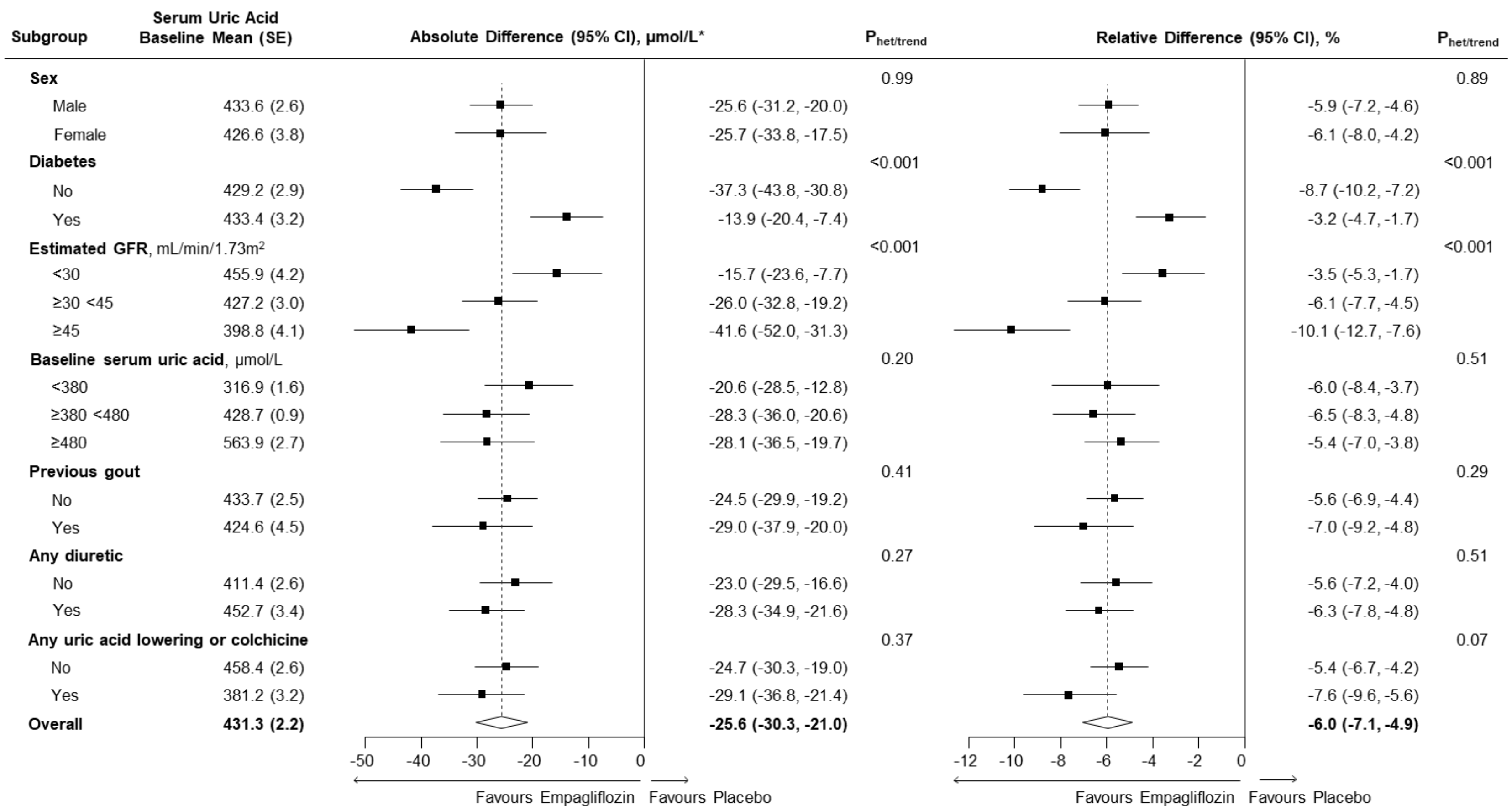

*To convert uric acid to mg/dL, divide by 59.48 (380 μmol/L ≈ 6.4 mg/dL; 480 μmol/L ≈ 8.1 mg/dL). Analysis required participants to have at least one measurement of uric acid during follow-up at 2 and/or 18 months (n=2691 participants). Absolute differences in study-average uric acid (empagliflozin minus placebo) are derived from a repeated measures mixed model (MMRM) adjusted for baseline serum uric acid (in continuous form), baseline-by-time interaction, the covariates used in the minimisation algorithm (categories of age, sex, diabetes, estimated glomerular filtration rate, urinary albumin-to-creatinine ratio and region) fixed categorical effects of time, treatment allocation and treatment-by-time interaction, and weighted in proportion to the amount of time between follow-up visits. Interaction terms are included in the MMRM models to assess for heterogeneity (sex, diabetes, previous gout, any diuretic therapy) between or trend (estimated GFR, serum uric acid) across subgroup-specific means and standard errors. Uric-acid lowering therapy includes xanthine oxidase inhibitors and primary uricosuric drugs (see Methods). GFR = glomerular filtration rate. *The figure reflects baseline use of diuretics (and other characteristics) however it is relevant that among those participants in the full trial cohort who were not taking a loop diuretic at randomization, 159 of 2453 (6.5%) in the empagliflozin group compared with 212 of 2409 (8.8%) in the placebo group started such medication during follow-up, representing a 26% lower likelihood of a new loop diuretic prescription among the empagliflozin group (risk ratio 0.74, 95% CI, 0.60 to 0.90). Missing baseline uric acid (4/2691 participants) was imputed with the baseline mean and so participants are included in the middle subgroup category ≥380 <480 μmol/L.

Supplementary Figure 5: Effects of empagliflozin versus placebo on serum uric acid by diabetes status and eGFR

Subgroup	Serum Uric Acid Baseline Mean (SE)	Absolute Difference (95% CI), µmol/L*	P_{trend}	Relative Difference (95% CI), %	P_{trend}
No diabetes eGFR <30	450.9 (5.7)	-23.2 (-34.7, -11.6)		-5.3 (-7.9, -2.6)	
No diabetes eGFR ≥30 <45	423.5 (4.2)	-37.1 (-46.7, -27.4)	<0.001	-8.8 (-11.0, -6.5)	<0.001
No diabetes eGFR ≥45	410.2 (5.2)	-56.6 (-70.2, -43.1)		-13.6 (-16.9, -10.3)	
Diabetes eGFR <30	460.2 (6.0)	-8.7 (-19.6, 2.2)		-1.9 (-4.4, 0.5)	
Diabetes eGFR ≥30 <45	430.7 (4.2)	-15.3 (-24.8, -5.8)	0.18	-3.6 (-5.8, -1.4)	0.14
Diabetes eGFR ≥45	382.9 (6.5)	-21.3 (-37.4, -5.3)		-5.3 (-9.3, -1.3)	
**Overall**	**431.3 (2.2)**	**-25.6 (-30.3, -21.0)**		**-6.0 (-7.1, -4.9)**	

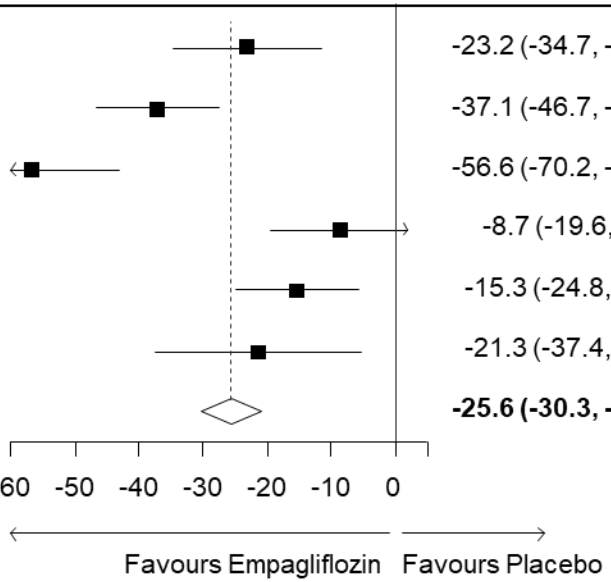

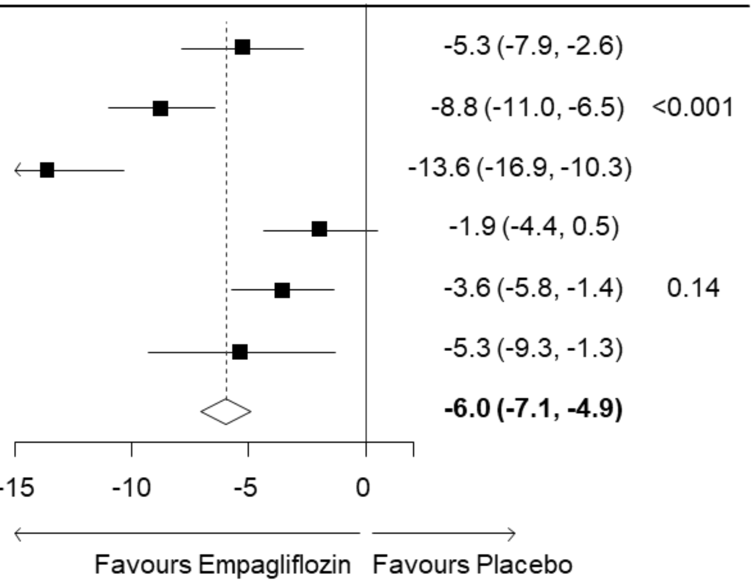

*To convert uric acid to mg/dL, divide by 59.48 (25.6 µmol/L ≈ 0.4 mg/dL). Analysis required participants to have at least one measurement of uric acid during follow-up at 2 and/or 18 months (n=2691 participants). Absolute differences in study-average uric acid (empagliflozin minus placebo) are derived from a repeated measures mixed model (MMRM) adjusted for baseline serum uric acid (in continuous form), baseline-by-time interaction, the covariates used in the minimisation algorithm (categories of age, sex, diabetes, estimated glomerular filtration rate, urinary albumin-to-creatinine ratio and region) fixed categorical effects of time, treatment allocation and treatment-by-time interaction, and weighted in proportion to the amount of time between follow-up visits. There was evidence that the trend in uric acid lowering effects across eGFR categories differed by diabetes status (likelihood ratio test P<0.001 comparing models without and with the additional interaction terms diabetes*eGFR*treatment and diabetes*eGFR *treatment*time with the categorical eGFR variable in continuous form), therefore interaction terms are included in the MMRM models to assess for trend across subgroup-specific means and standard errors in estimated GFR categories in participants with and without diabetes separately. eGFR = estimated glomerular filtration rate. Missing baseline uric acid (4/2691 participants) was imputed with the baseline mean.

Supplementary Figure 6: Further exploration of differential effects of empagliflozin on serum uric acid in participants with and without diabetes

Subgroup	Baseline Uric Acid, µmol/L* Mean (SE)	Baseline eGFR, mL/min/1.73m² Mean (SE)	% on insulin at baseline	Absolute Difference (95% CI)	$P_{het/trend}$
**Diabetes status**					<0.001†
No diabetes	429.2 (2.9)	38.0 (0.4)	-	-37.3 (-43.8, -30.8)	
Diabetes not on insulin	421.3 (4.4)	37.4 (0.5)	-	-20.3 (-29.8, -10.8)	
Diabetes on insulin	444.3 (4.5)	34.2 (0.4)	100.0	-8.0 (-17.0, 0.9)	
**HbA1c**, mmol/mol					<0.001‡
<39	426.7 (3.5)	38.0 (0.5)	2.7	-31.1 (-38.9, -23.4)	
≥39 <48	433.1 (3.9)	36.2 (0.4)	15.3	-38.1 (-46.1, -30.1)	
≥48 <58	430.2 (5.4)	37.1 (0.7)	52.1	-17.1 (-28.5, -5.7)	
≥58	438.9 (5.4)	35.5 (0.6)	74.4	5.1 (-6.4, 16.6)	
**Overall**	**431.3 (2.2)**	**36.9 (0.3)**	**26.2**	**-25.6 (-30.3, -21.0)**	

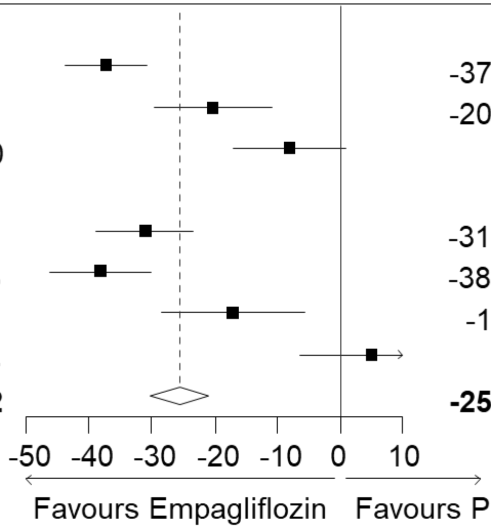

*To convert uric acid to mg/dL, divide by 59.48 (431.3 µmol/L ≈ 7.2 mg/dL; 25.6 µmol/L ≈ 0.4 mg/dL). Analysis required participants to have at least one measurement of uric acid during follow-up at 2 and/or 18 months (n=2691 participants). Absolute differences in study-average uric acid (empagliflozin minus placebo) are derived from a repeated measures mixed model (MMRM) adjusted for baseline serum uric acid (in continuous form), baseline-by-time interaction, the covariates used in the minimisation algorithm (categories of age, sex, estimated glomerular filtration rate, urinary albumin-to-creatinine ratio and region) fixed categorical effects of time, treatment allocation and treatment-by-time interaction, and weighted in proportion to the amount of time between follow-up visits. Interaction terms are included in the MMRM models to assess for †heterogeneity or ‡trend across subgroup-specific means and standard errors. Participants with missing HbA1c (32/2691, 1.2%) are included in the ≥39 <48 mmol/mol category containing the mean value of 46 mmol/mol. eGFR = estimated glomerular filtration rate; HbA1c = glycated haemoglobin. Missing baseline uric acid (4/2691 participants) was imputed with the baseline mean.
